# Carbon Nanodots in Photodynamic Antimicrobial Therapy: A Review

**DOI:** 10.3390/ma13184004

**Published:** 2020-09-10

**Authors:** Rachael Knoblauch, Chris D. Geddes

**Affiliations:** Institute of Fluorescence and Department of Chemistry and Biochemistry, University of Maryland, Baltimore County, 701 East Pratt Street, Baltimore, MD 21202, USA; rknobla1@umbc.edu

**Keywords:** carbon dots, carbon nanodots, carbon quantum dots, photosensitization, antimicrobial photodynamic therapy, antibacterial, photocatalytic disinfection, antimicrobial materials

## Abstract

Antibiotic resistance development in bacteria is an ever-increasing global health concern as new resistant strains and/or resistance mechanisms emerge each day, out-pacing the discovery of novel antibiotics. Increasingly, research focuses on alternate techniques, such as antimicrobial photodynamic therapy (APDT) or photocatalytic disinfection, to combat pathogens even before infection occurs. Small molecule “photosensitizers” have been developed to date for this application, using light energy to inflict damage and death on nearby pathogens via the generation of reactive oxygen species (ROS). These molecular agents are frequently limited in widespread application by synthetic expense and complexity. Carbon dots, or fluorescent, quasi-spherical nanoparticle structures, provide an inexpensive and “green” solution for a new class of APDT photosensitizers. To date, reviews have examined the overall antimicrobial properties of carbon dot structures. Herein we provide a focused review on the recent progress for carbon nanodots in photodynamic disinfection, highlighting select studies of carbon dots as intrinsic photosensitizers, structural tuning strategies for optimization, and their use in hybrid disinfection systems and materials. Limitations and challenges are also discussed, and contemporary experimental strategies presented. This review provides a focused foundation for which APDT using carbon dots may be expanded in future research, ultimately on a global scale.

## 1. Introduction: Photodynamic Antimicrobial Therapy and Carbon Nanodots

Although the threat of antibiotic resistance development in pathogenic bacteria is not a new challenge, it remains to date a pressing concern in regard to global health. Antibiotics have been a primary tool for combating infectious diseases, yet the timescale of development for next-generation antibiotics remains lengthy due to the complexities and cost of not only drug discovery, but also clinical testing and ultimately approval. Additionally, bacteria have a multitude of mechanisms by which they can rapidly acquire resistance [1,2], which is of particular concern for high-risk populations such as those in healthcare settings [3,4]. For those with concurrent conditions, such as cancer, bacterial infections can severely worsen patient health [5,6]; infections may also delay wound recovery even when treated, with some negative health impacts associated with current disinfection techniques [7]. These cumulative effects place a premium on developing techniques to mitigate the threat of infection from deadly pathogens, namely by improved sterilization technologies. A selection of current disinfection strategies include spray disinfectants containing harsh chemicals, ultra-violet radiation that is deleterious to human health, and antimicrobial nanotechnology—for example silver nanoparticles—which have an inherent antimicrobial character that is not controllable except by dose/exposure.

**Figure 1:**
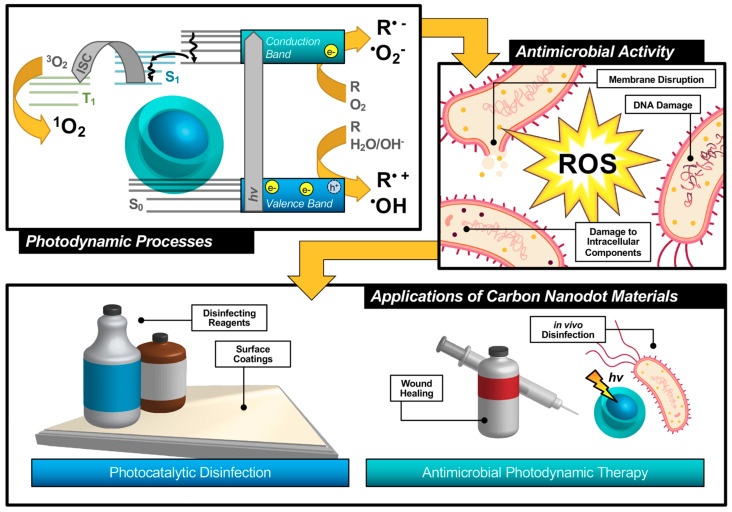
Schematic description of antimicrobial photodynamic therapy and photocatalytic disinfection using carbon nanodots.

An alternative technique known as antimicrobial photodynamic therapy (APDT) [8,9,10]—or for variations in application: antimicrobial photodynamic inactivation (APDI) [11] of bacteria or photocatalytic disinfection [12]—presents an intriguing option for a different class of disinfection materials. This process is based in the photodynamic response of a photosensitizing agent, as shown in the first step illustrated in Figure 1. As shown by the simplified Jablonski diagram, incident light induces an excitation event in the photosensitizer (S_0_ → S_1_); from this stage, the excited electron may undergo a spin flip in a process known as intersystem crossing (ISC, S_1_ → T_1_) to form a triplet excited state. Occupation of this state permits triplet-triplet interactions with ground-state molecular oxygen; energy transfer, in this case, produces highly reactive singlet oxygen species (ROS), for example, singlet oxygen (^1^O_2_), as a result of a “Type II” photosensitization mechanism [13,14].

Photosensitizers such as carbon nanodots (CNDs), which will be the subject of this review, are known to exhibit fluorescence due to the generation of electron/hole pairs (e^−^/h^+^), also shown in the first step of Figure 1. Either of these (e^−^ or h^+^) can undergo electron transfer, known as Type I photosensitization, with different proximal species such as water, molecular oxygen, or organic agents in solution to produce radical species, including hydroxyl radical (^•^OH) and superoxide anion radical (O_2_^•−^) to name a few [14]. Both Type I and II mechanisms result in highly reactive ROS and are known to inflict severe oxidative damage on microbial cells as illustrated in Figure 1 [15]. Destruction may occur at the membrane causing morphological changes or cytoplasmic leakage; additionally, diffusing ROS can cause DNA impairment or damage to other intracellular components, such as ribosomes. Oxidative damage from ROS has been of increasing interest in the context of antibiotic-resistant bacteria, as it proves more complex for bacteria to develop resistance to this method [16,17]. Additionally, there is no known enzyme to detoxify ^•^OH and ^1^O_2_ [18]. Furthermore, studies have shown that APDT may be tuned to permit high spatial control, thereby reducing deleterious impacts on nearby mammalian cells during treatment [17,19]. Alternatives such as photothermal therapies have promise but do not share the spatial resolution possible with APDT [20]. Accordingly, an emphasis has been placed on optimizing the properties of photosensitizing molecules for antimicrobial applications, particularly in tuning the photophysical properties of those agents [21] for maximized ROS quantum yields [22,23]. By optimizing these characteristics, it is possible to improve the efficiency of light utilization by the photosensitizer, and even to select for activation wavelengths that are themselves not inherently harmful, unlike short-wavelength ultraviolet light (UV-C) [24]. In contrast, TiO_2_ materials, which are commonly used for photocatalytic disinfection applications, suffer from low utilization of solar energy [22,25] and require UV activation [26,27]. Beyond quantum efficiency, it is also desirable to tune the proximity or adherence of the photosensitizer to bacterial cells [21]. This is of particular importance since ROS are by nature highly reactive; to prevent off-target interactions, the species must be generated locally to the cell of interest. Beyond these properties, an ideal APDT photosensitizer would also have a dynamic scaffold structure that would permit facile adaptation for multi-modal purposes. This could include modification for varied delivery platforms, changing surface structures to achieve solubility in different disinfectant reagents, or even covalent attachment for antimicrobial surface coating materials, as in photocatalytic disinfection (Figure 1). Dynamic structures could also improve theranostic applications, permitting simultaneous imaging/detection in the context of wound healing or in vivo disinfection therapies (Figure 1). Beyond medical therapies, applications could be found in food safety, water disinfection, and solar-driven disinfection [3,25,28]. Ultimately, an inexpensive and sustainable photosensitizer would be optimal for a broad-use, up-scaled commercial APDT photosensitizer.

Although numerous small molecule photosensitizers have been developed for APDT, these are limited in commercial scalability due to complex syntheses, high cost, and purification requirements. Recently, carbon nanodots have received increasing attention for use as a nanoparticulate photosensitizer for APDT and photocatalytic disinfection. These fluorescent nanoparticles are known to be a combustion byproduct and/or the product of incomplete carbonization from biomass, resulting in quasi-spherical, fluorescent particles less than 10 nm in size with broad-spectrum absorption profiles. Due to their synthetic sources, these particles present a potentially inexpensive and “green” option for a scalable photocatalytic disinfection material. Other recent reviews have focused on carbon nanodots in general as antimicrobial agents [29,30,31,32]; herein, we present and discuss recent developments surrounding the application of carbon nanodots in antimicrobial photodynamic therapy.

## 2. Characterization of Carbon Nanodots: An Overview of Key Spectroscopic/Physical Properties and Analysis Methods

**Figure 2:**
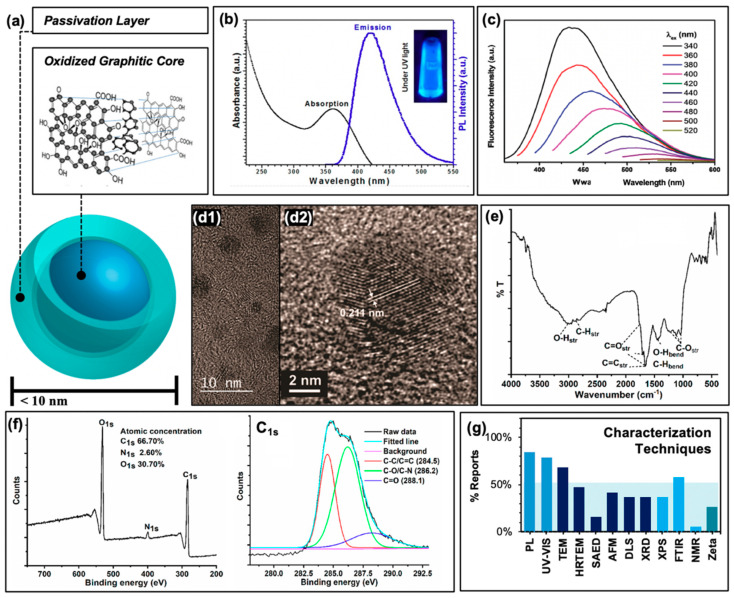
Characterization of carbon nanodots. (**a**) Schematic of “core-shell” structure of surface passivated carbon nanodots. *Inset* displays an example oxidized graphitic layer comprising the nanodot core. (**b**) Typical photoluminescence characteristics. (**c**) Excitation-dependent emission from carbon nanodots at multiple excitation wavelengths. (**d1**) Transmission electron micrograph (TEM) of carbon dots and (**d2**) high-resolution (HRTEM) image displaying typical graphene lattice spacing. (**e**) Typical FTIR spectrum. (**f**) Example X-ray photoelectron spectrograph (XPS, *left*) and high-resolution scan of C1s spectrum *(right*). (**g**) Summary of the characterization techniques reported by the references cited in this work. **Abbreviations:** PL (photoluminescence), SAED (selected area electron diffraction), AFM (atomic force microscopy), DLS (dynamic light scattering), XRD (X-ray diffraction), FTIR (Fourier transform infrared spectroscopy), NMR (nuclear magnetic resonance), “Zeta” (zeta potential). **Permissions**: (**a**) Inset adapted from Reference [35]—Published by The Royal Society of Chemistry. (**b/c**) Reprinted with permission from (**b**) Reference [22], Copyright © 2019 American Chemical Society and (**c**) Reference [19], Copyright © 2017, John Wiley and Sons. (**d**) Adapted with permission from Reference [36], Copyright © 2018, American Chemical Society. (**e**) Reprinted and (**f**) adapted with permission from (**e/f**) Reference [3], Copyright © 2019, American Chemical Society.

Other carbon-based structures, for example fullerenes or graphene quantum dots, have been known for some time to generate reactive oxygen species and have found applications in anti-cancer photodynamic therapies [33,34]. Carbon nanodots, also referred to as “carbon dots” or “carbon quantum dots”, differ from these in both their chemical structure and fluorescent properties. Regarding the latter, carbon dots in general are composed of a core consisting of many-layered sheets of oxidized graphene [24,35], as depicted schematically in Figure 2a. Each sheet varies in the extent of its ring system and pi conjugation, resulting in a quasi-spherical morphology after these sheets have assembled [36]. An additional passivation layer of organic nature is frequently functionalized to the graphitic core to achieve desirable properties; these may include reduced particle aggregation, optimized solubility, or an improved quantum yield to name a few. In total, this structure is on the order of less than 10 nm in size. Typical absorption and sample emission spectra are shown in Figure 2b, demonstrating the strong UV absorption and visible absorption tail inherent to carbon dot structures; studies have been conducted to further extend the absorption of carbon dots into the visible or even NIR region [37,38,39]. Carbon dots are also known to be capable of up-conversion photoluminescence [27,40] which will be discussed further in the context of antimicrobial applications in Section 4.2. Beyond synthetic optimization of excitation/emission properties, carbon dot samples also uniquely have an excitation-dependent emission profile, as shown in Figure 2c. This allows, to a degree, luminescence tuning simply by adjusting the excitation source used in an experiment; however, varying carbon dot quantum efficiencies at each wavelength must be carefully considered. Studies have indicated exciton or electron/hole (e^−^/h^+^) recombination, surface trap states, and quantum confinement effects to all contribute to carbon dot fluorescent properties [41,42,43]. Studies have also demonstrated that excitation of the carbon dot core, rather than surface states, results in different observed photodynamic properties [44].

It must be admitted, however, that these properties can vary widely from group to group, as numerous synthesis methods for carbon dots have been established and employed. These include both “top-down” and “bottom-up” approaches [45]. In the former, a “bulk” scale carbon structure—such as biomass, for example—is converted to carbon nanodots through methods such as arc discharge, laser ablation, or electrochemical stress to name a few. For “bottom-up” strategies, small molecule precursors are used instead in microwave-assisted, hydrothermal, or thermal techniques [45]. An example of this is the combustion-based collection of carbon nanodots from methane gas, established by Schmitz et al. from our laboratory [44]. This strategy has been further adapted by us to achieve carbon dot structures of different photophysical characteristics [46,47]; the results of some of these studies will be discussed herein. Although many of the aforementioned synthesis methods can be found in the literature, hydrothermal treatment in either a top-down or bottom-up regime is most commonly employed. In particular, this method is favored amongst reports of carbon dot “green syntheses” from biomass, or natural precursors [17,22,48,49].

In order to classify carbon dots as such, researchers typically characterize their resulting structures by a number of methods. Included with the photophysical characterization mentioned earlier, size determination is common and necessary; this is typically done by transmission electron microscopy (TEM) and/or high-resolution TEM (HRTEM) as shown in Figure 2d, although other methods such as atomic force microscopy (AFM) and dynamic light scattering (DLS) are also employed. TEM images typically reveal the <10 nm sizes mentioned previously (Figure 2d2); ideally, a combination of TEM and AFM helps to confirm the quasi-spherical nature of the carbon dots studied. Further, HRTEM reveals graphitic lattice spacings such as those shown in Figure 2d2. Additional techniques such as selected area electron diffraction (SAED, an extension of TEM technologies) and X-ray diffraction (XRD) may also be employed to probe the crystallinity of the samples, although these are less common in the carbon dot literature. To understand the chemical composition of carbon dot samples, techniques such as Fourier-transform infrared spectroscopy (FTIR, Figure 2e) and X-ray photoelectron spectroscopy (XPS, Figure 2f) are frequently used to reveal functional groups, elemental composition, and covalent bonding patterns within the carbon dot structures. To a lesser degree, nuclear magnetic resonance (NMR) is also applied, although as expected the results of this analysis can prove difficult to decode given the high number of hydrogens contained within a single carbon dot structure, and the wide variety of local environments surrounding these hydrogens. A summary of all of these characterization techniques and their relative rate of use in the selection of references examined (namely, those reporting applications in APDT) is presented in Figure 2g. It must be noted also that for some of these studies, additional characterization is included in previous publications that are then cited in subsequent reports, for example in the case of photoluminescence characterization; these are not included in the data for Figure 2g. 

One technique of particular note which has not yet been discussed is the carbon dot zeta potential measurement. When reported, this is typically given as a way to characterize the relative surface charge of a carbon dot sample. Although not widely included in the general characterization of carbon dots, this is a consideration of extreme importance in the discussion of carbon dots as APDT photosensitizers. Firstly, the zeta potential measurement can give some insight into the physical stability of carbon dots, namely the tendency of structures to aggregate under different pH environments. Perhaps more important, however, is that both Gram-negative and -positive bacteria exhibit negative charges on their exterior cell walls, although these properties are different. The zeta potential subsequently provides an important indicator of potential cell/carbon dot electrostatic interactions and resulting APDT efficiency; in fact, a recent study by Verma et al. demonstrates using zeta potential analysis that positive/neutrally-charged carbon dots exhibit higher antibacterial activity against *Escherichia coli* (*E. coli*) than dots carrying a negative potential [50]. Using this characterization technique, researchers may more effectively tune the surface passivation properties of their carbon dots for expanded applications. In fact, the structural, chemical, and photophysical characteristics of carbon dots imbue a strong versatility in their potential through tuning and modification. Accordingly, carbon dots are mentioned to have been employed for photocatalytic dye degradation [22,27,40,51], photocatalytic/electrical water splitting [22,52], solar devices [22,40,53], detection and biosensing [3,16,27,51], drug delivery [3,27,51], bioimaging [3,17,27,51], gene transmission [3,17], and even in LED technologies [27,54]—although this list is far from exhaustive and is ever-expanding, including APDT applications.

## 3. Carbon Nanodots as Photodynamic Antimicrobial Agents

### 3.1. Generation and Detection of Reactive Oxygen Species from Carbon Nanodots

**Figure 3:**
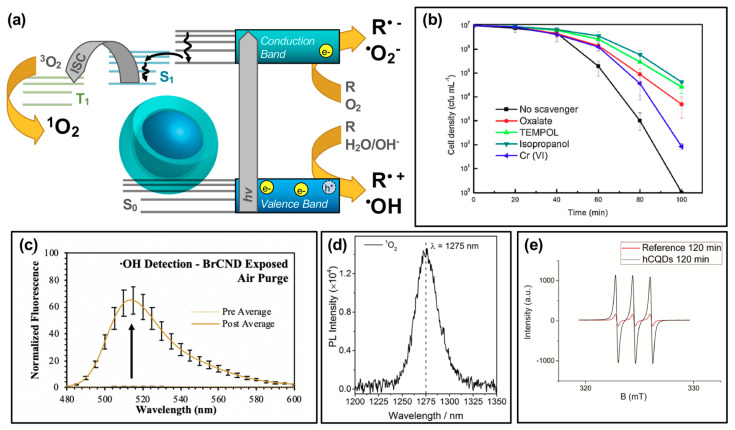
Reactive species generation from photodynamic activation of carbon nanodots. (**a**) Schematic for the generation of reactive species following an excitation event. (**b**) *E. coli* cell viability (density) after photodynamic treatment with a CDs/WO_3_/Na_2_W_4_O_13_ composite, with varying response due to the application of scavenging reagents for various reactive species. These reagents include oxalate (e^−^ scavenger), TEMPOL (•O_2_^−^ scavenger), isopropanol (•OH scavenger), and Cr(VI) for scavenging h^+^. (**c**) Detection of hydroxyl radical (•OH) using fluorescence-on probe hydroxyphenyl fluorescein (from Ref. [56]). (**d**) Singlet oxygen (^1^O_2_) luminescence direct detection from activated carbon dots. (**e**) Detection of singlet oxygen using electron paramagnetic resonance (EPR) spectroscopy. For additional details regarding the reported studies, the reader is referred to Table 1 of the main text. **Abbreviations:** ISC (intersystem crossing), PL (photoluminescence), hCQDs (hydrophobic carbon quantum dots), BrCND (brominated carbon nanodots). **Permissions**: (**b**) Reprinted from Journal of Hazardous Materials, 359, Zhang, J. et al., “Carbon dots-decorated Na_2_W_4_O_13_ composite with WO_3_ for highly efficient photocatalytic antibacterial activity”, 1–8, Copyright © 2018 with permission from Elsevier [12]. (**d**) Adapted with permission from Reference [55]. Copyright © 2018, American Chemical Society. (**e**) Adapted with permission from Reference [36]. Copyright © 2018, American Chemical Society.

Carbon dots have recently seen more focus as potential APDT photosensitizers, with publications reporting the generation of reactive oxygen species in general from excited structures [3,16,19,25,28,55]. To better understand the specific species generated and thereby efficiently tune the carbon dot structures to yield desired species, researchers have conducted further investigation to determine if carbon dots undergo both Type I and Type II photosensitization mechanisms, schematically reiterated in Figure 3a. In a study by Zhang et al., the Type I process was probed by examining cell viability following photosensitization with carbon dots hybrid structures (indicated as CDs/WO_3_/Na_2_W_4_O_13_ nanocomposite) and various reactive species scavengers (Figure 3b) [12].

These species are generated by an electron transfer process occurring upon the excitation of carbon dots and the formation of e^−^/h^+^ pairs [22,28,51]. Using *E. coli*, the group found that the bacterial inactivation efficiency of the nanocomposite could be partially mitigated by all of the scavengers tested, including sodium oxalate, 4-hydroxy-TEMPO (“TEMPOL”), isopropanol, and potassium dichromate. This suggested that the overall antibacterial effect was due to not one, but multiple Type I photosensitization products generated including h^+^, O_2_^•−^, ^•^OH, and e^−^ respectively. It should be noted here that in this particular study, the carbon dots themselves were not inherently responsible for the generation of the indicated reactive species; however, their incorporation into the nanocomposite structure is critical to observe a photodynamic disinfection effect relative to the Na_2_W_4_O_13_/WO_3_ control structure. Additional experimental details for this study, and other studies referenced by the figures in this section, may be found in Table 1. Further discussion of hybrid materials will be the focus of Section 4. Type I photosensitization products have also been identified from other carbon dot structures, whereby the intrinsic characteristics of the dots themselves are responsible for the generation. In a study by us, ^•^OH was detected from a brominated carbon nanodot structure collected via a combustion-based method (Figure 3c) [56]. Using the fluorescence-on probe hydroxyphenyl fluorescein (HPF), the species was detected by a fluorescence increase following photosensitization steps. Other studies have similarly reported Type I photosensitization products from carbon dots, including both ^•^OH as discussed [25], and O_2_^•−^ [25,55]. It is intriguing to note also that in some cases, such as a 2018 study by Zhang et al. [55], one Type I product is detected while the other is not (in their case, ^•^OH was not detected by their methods). Type I products are in fact quite interconnected, potentially inter-converting rapidly as illustrated in the following reaction schematic [51]:

CNDs + *hv* → e^−^ + h^+^, (R1)

O_2_ + e^−^ → O_2_^•−^, (R2)

H_2_O + h^+^ → H^+^ + ^•^OH, (R3)

O_2_^•−^ + H^+^ → ^•^O_2_H, (R4)

H^+^ + ^•^O_2_H → H_2_O_2_, (R5)

H_2_O_2_ + e^−^ → OH^−^ + ^•^OH, (R6)

OH^−^ + h^+^ → ^•^OH, (R7)

Reaction of these species, dependent upon the generation environment, may clearly influence not just which species is present, but also if downstream ROS such as the hydroperoxyl radical (^•^O_2_H) and hydrogen peroxide (H_2_O_2_) are produced. It should be noted that although we frequently consider ROS as being oxygen-dependent, hydroxyl radical may also be produced by Reaction (R3) or (R7), permitting the generation of ROS in hypoxic aqueous environments [19].

The Type II photosensitization product known as singlet oxygen has also been investigated as a potential product from carbon nanodot photosensitizers [17,25,36,55,56], shown in the simplified Jablonski diagram of Figure 3a. In this regime, carbon dots can be considered as small molecule fluorophores; triplet character of the carbon dots is therefore a strong factor in the ability of singlet oxygen to be generated. While the long lifetime inherent of triplet excited states provides a larger window of opportunity for diffusing oxygen to interact with the photosensitizer, thereby undergoing energy transfer and generating singlet oxygen, there is also the possibility that other diffusing species or even competitive radiative decay pathways such as alpha/delayed fluorescence may deactivate the excited triplet state without the production of singlet oxygen. Nonetheless, singlet oxygen has been detected by carbon dot photosensitization. In a study by Zhang et al., singlet oxygen was directly detected from their carbon dot structures by measuring the phosphorescent emission of singlet oxygen, observable at ~1270–1275 nm (Figure 3d) [55]. Singlet oxygen has also been detected from brominated carbon nanodots using the singlet oxygen specific fluorescence-on probe Singlet Oxygen Sensor Green™ [56], and by electron paramagnetic resonance measurements, as shown for hydrophobic carbon quantum dots (hCQDs) from a study by Stankovic et al. in Figure 3e [36]. These authors additionally measured the quantum yield of the hCQDs using tetraphenylporphyrin as a standard, arriving at a value of 0.31. They note that although this does not match the efficiency of fullerenes (0.86), carbon dots themselves do not quench singlet oxygen and therefore are a promising scaffold to adapt and apply as a photodynamic antimicrobial agent [36]; this is advantageous versus the Type I mechanism, as carbon dots have been shown to behave in some instances as radical scavengers with antioxidant behavior [57]; it should also be noted that unlike Type I products, which can result from charge transfer to a variety of reactants, singlet oxygen generation is dependent specifically upon the availability of oxygen in the system, with reported loss of detection under oxygen-depleted conditions being shown in several studies for carbon dot materials [36,56].

It should be noted that the figures of this review present publication results in the context of single data sets, however, in most cases the detection of respective ROS is validated by additional experimental strategies. These may have been conducted for carbon dots either in an in situ or solution-based context, whereby bacterial cells do or do not play a role in the experimental detection respectively. For intracellular general ROS detection, ROS/RNS assay kits may be used [58], and/or fluorescence-on probes such as dihydrorhodamine 123 (DHR 123) [3,16,59] or dichlorofluorescein-diacetate (DCFH-DA) [25]. In cell-less, solution-based detection, DCFH can also be used, although without the diacetate [19]. Alternatively, probes such as 1,3-Diphenylisobenzofuran (DPBF) have also been employed [5,25]. Detection of specific species has been performed in multiple studies using the techniques summarized in Figure 3, including ROS detection by scavenging reagents [12,25,55], by directly detecting singlet oxygen luminescence [36,55], by using EPR (or ESR) [12,25,36,55], and through the utilization of fluorescence-on [56,60] or colorimetric probes [17,55].

In addition to the antibacterial effects that will be discussed in the subsequent section, ROS detected from carbon dots and related graphene quantum dots have also been noted to cause lipid peroxidation [25], depletion of antioxidants in cells [25], and even to a degree dye degradation [49]. The secondary effects of ROS generated by carbon dots remain to be further elucidated and should persist as a consideration from both a fundamental and experimental perspective for future carbon dot APDT investigations. Not only do they reveal potential mechanisms of cellular destruction by carbon dots, they also present ways in which the technology may be further improved regarding efficiency, longevity, and stability.

### 3.2. Photoinduced Antimicrobial Activity from Carbon Nanodots

**Figure 4:**
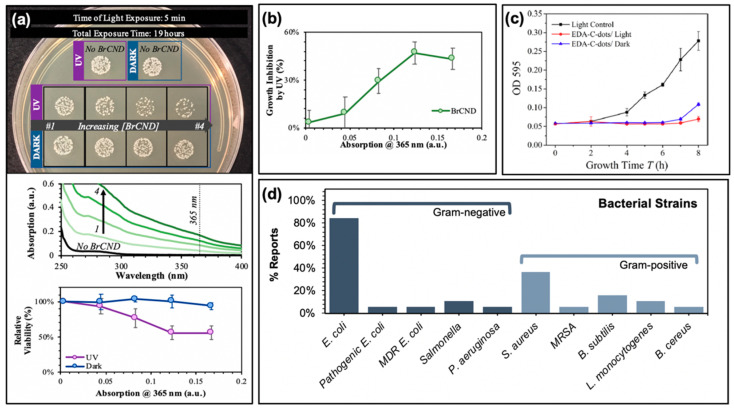
Photodynamic antimicrobial characteristics of carbon nanodots. (**a**) *Top*—Concentration-dependent growth reduction of *Staphylococcus aureus* exposed to brominated carbon nanodots (BrCND). *Middle*—Absorption spectra for BrCND of varying concentrations, corresponding to solutions used in top panel. *Bottom*—Relative viability of bacterial samples under both dark and light-exposed conditions (from Reference [56]). (**b**) Photodynamic inhibition of bacterial growth from (**a**) (from Reference [56]). (**c**) Bacterial growth curves of *Escherichia coli* samples after photodynamic treatment with carbon nanodots (EDA-C-dots). (**d**) Summary of investigated bacterial strains in the references reported by this review. For additional details regarding the reported studies, the reader is referred to Table 1 of the main text. **Abbreviations:** CDs (carbon nanodots). **Permissions**: (**c**) Adapted with permission from Reference [26]. Copyright © 2016, American Chemical Society.

Studies reporting the generation of reactive oxygen species from carbon dots have also by extension recognized and investigated the potential for antimicrobial effects as well. In fact, carbon dots have been shown to exhibit antibacterial efficiencies comparable or even superior to common small-molecule photosensitizers [55]. Examples of antibacterial effects from carbon dot photosensitization can be found in Figure 4. In a recent report by our lab, the antimicrobial effects of brominated carbon nanodots from photosensitization by UVA light (long wavelength, 365 nm) were studied using Gram-positive *Staphylococcus aureus* as a model bacterium (Figure 4a,b). Previous reports had characterized brominated carbon nanodots (BrCNDs) to be weakly fluorescent, with a phosphorescent signal emerging following bromination that was otherwise unobserved in the non-bromine-containing carbon dots collected by the same combustion-based method [46]. This phosphorescent signal was found to be pH-dependent, detectable only in acidic environments; at higher pH, fluorescence intensities were restored, and phosphorescence was lost. The detection of phosphorescence confirmed triplet character from the BrCNDs, and singlet oxygen was detected using the Singlet Oxygen Sensor Green™ fluorescence-on probe, alongside the Type I product already shown in Figure 3c [56,60]. Subsequent in situ experiments with *Staphylococcus aureus* (*S. aureus*) further confirmed the bactericidal capabilities of the BrCNDs; interestingly, the bacteria were not susceptible to significant growth reduction due to pH alone (all results in Figure 4a conducted at a pH of ~3, control studies in Reference [56]). With BrCND incorporation, as shown in Figure 4a, viability loss was seen as a result of BrCND excitation. Similar to the pH control (“no BrCND”), samples containing the equivalent concentrations of BrCND with *no* excitation (“dark”) had no substantial loss in relative viability. The photosensitization effect for BrCND can be observed clearly in Figure 4b, with increasing absorption values of BrCND corresponding to different dilutions of the samples [56]. In this case, no dark toxicity effects were observed for the concentration range of BrCND tested, as is common for other carbon dot photosensitization studies [3,16,17,19,22,49,51,55]. It must be noted, though, that it is not always true that carbon dots alone exhibit no toxicity to bacterial cells *independent* of a photosensitization mechanism [3,25,26,28,36]. Figure 4c illustrates such a case; in a study by Meziani et al., EDA-passivated carbon dots (EDA-Cdots) significantly inactivate bacterial growth, as evidenced in the growth curve shown (monitored at 595 nm bacterial sample absorption) [26]. This is true for both the dark and light-exposed conditions, although the bacterial samples without photosensitization showed a lengthened lag time by approximately 1 h, as compared to the dark sample. The authors attributed the similarity in growth curves to inadequately optimized experimental parameters, and in fact, other studies reported in the same publication demonstrate a lack of toxicity from carbon dots under dark conditions, whereas the exposed results appear significantly different. It cannot be ignored, however, that there are known sources of toxicity from carbon dot structures interacting with bacterial cells.

These effects are discussed in a review by Anand et al. [31] and have been mentioned by others [61,62,63,64,65,66]; they include cell well disruption [61,62,63,64] and DNA/RNA damage [61,65,66] to name a few. Nonetheless, studies demonstrate significant bactericidal capabilities from photosensitization using carbon dots; strains studied by the reports summarized herein are compiled in Figure 4d, including multi-drug resistant (MDR) *E. coli* for one study and methicillin-resistant *Staphylococcus aureus* (MRSA) as well [19].

**Figure 5:**
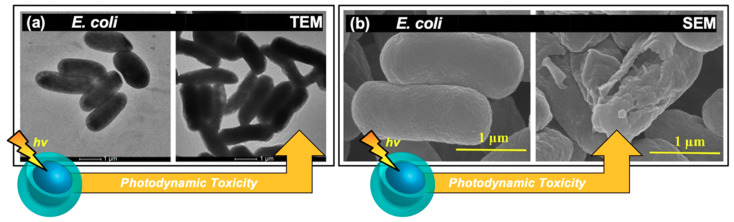
Morphological changes in bacterial cells exposed to carbon nanodot photosensitizers. (**a**) Transmission electron microscopy (TEM) images of *E. coli* cells after (*left*) light exposure only and (*right*) after light-exposure in carbon dot treated samples. (**b**) Scanning electron microscopy images displaying *E. coli* cells both (*left*) pre and (*right*) post exposure for carbon dot treated samples. For additional details regarding the reported studies, the reader is referred to Table 1 of the main text. **Permissions**: (**a**) Adapted from Reference [16]. Open access. (**b**) Adapted from Reference [49], open access (licensed under Creative Commons Attribution 4.0 International License: http://creativecommons.org/licenses/by/4.0/).

In some instances, morphological changes were also examined in conjunction with the viability studies from colony growth, disk diffusion assays, and/or growth curves. Morphological changes in fact are expected as part of a photosensitization mechanism, as ROS have been shown to act upon and severely compromise bacterial membranes; carbon dots specifically have been noted to induce lipid peroxidation, as mentioned previously. Examples of this phenomenon are shown in Figure 5, reported both by TEM (Figure 5a) and scanning electron microscopy (SEM, Figure 5b) for different studies. In the former case (Figure 5a), *E. coli* cells were exposed to visible light for one hour, both with and without 5 μg/mL of carbon nanodot sample in a 2018 study by Dong et al. [16]. Interestingly, when examined in terms of colony growth, the carbon dot sample alone at this concentration was insufficient to induce cell death under photosensitization conditions. Only at higher concentrations (25+ μg/mL) were antibacterial photosensitization effects observed. Yet upon examination of the TEM images, it is clear that even at low concentrations the carbon dot samples begin to have deleterious effects on bacterial morphology. In a report by Kavitha et al., the morphology of *E. coli* cells was also studied, this time using SEM (Figure 5b) [49]. In this case, cells were treated with 70 μg/mL of carbon dots; the left-hand image displays the cells before photodynamic treatment, while the right-hand image displays the effects after a 10 h exposure period with room light. Significant differences in morphology are observed here, including membrane wrinkling and even rupture. Although the 10 h exposure interval shown here does in fact seem quite lengthy, the authors do additionally note a significant impairment of growth (monitored by OD590) after as little as 1 h exposure compared to the untreated control. Morphology changes have also been reported for Gram-positive bacteria treated with carbon dot photosensitizers including *S. aureus* [51].

Further, dark effects on morphology have been reported for carbon dot-treated *E. coli* cells with clear shrinkage/wrinkling observable in the SEM images, although these effects were only observable after nearly double the time at which morphological changes were detected for the visible light-treated samples [28].

It is noteworthy that to this point, and in fact as summarized in Figure 4d, the main testing of APDT from carbon dots has been conducted against “model” bacterial strains—that is, commonly used bacterial strains which may or may not have direct clinical relevance. As more research is conducted, a broader array of microorganisms should be explored in order to confirm the practical relevance of carbon dot photosensitizers. *Listeria monocytogenes*, for example, is a common pathogen associated with food-borne illnesses and could be a focus of food safety disinfection developments; to date, however, it has been only minimally researched [51,56]. Further, pathogenic and antibiotic-resistant strains should be applied more readily in the literature, as these are the main global health challenge and to date have received little attention [17,19,26]. It should be noted also that the efficacy of carbon dot photosensitizers regarding biofilms has been investigated only to a minor degree, however, biofilm formation is a major challenge in sterilization and disinfection. In a study by Stankovic et al., the anti-biofouling properties of hCQD Langmuir–Blodgett (LB) thin films were studied using *Bacillus cereus* (Gram-positive) and *Pseudomonas aeruginosa* (Gram-negative) strains [36]. The authors studied the antibacterial effects of hCQD LB films both on free cells and biofilms for each strain; for the latter, authors permitted biofilm growth prior to blue light exposure. Following irradiation for 2 h, the metabolic activity (and by extension, the viability of the cells) of *B. cereus* were significantly reduced; by comparison, the viability of *P. aeruginosa* was reduced to a modest degree [36]. The difference in observed antibiofouling effects could have numerous sources; namely, carbon dot interactions with various biofilms needs to be better understood. Further, diffusional capabilities of both carbon dots and ROS must be examined. Although an intriguing result, further research into the interaction of carbon dot photosensitizers and varying biofilms remains necessary.

An additional challenge is the seemingly contrary results reported for different studies in the literature examining the same bacteria; for example, from the data discussed herein it is clear that *E. coli* is susceptible to APDT from carbon dots, yet negative results have also been reported using *E. coli* [51]; the same may be said for *Samonella* [51,55]. That these conflicting results exist point to the need for a methodical examination of varied carbon dot structural, chemical, and photophysical properties, such that it is possible to achieve a comprehensive understanding of how these properties may be tuned to influence efficiency and specificity for particular bacterial cell types or strains. Research in this regard has begun, although not in the context of photodynamic carbon dot structures; in a 2016 study by Yang et al., it was found that by incorporating hydrophobic carbon chains and positively-charged, quaternary ammonium substituents, selectivity for Gram-positive bacteria could be obtained [67]. This was further confirmed in 2019 in a study by the same group [68]. Although in a “dark toxicity” context, these studies give insight to how carbon dots may be tuned for selectivity. In a related topic, Gao et al. demonstrated that their carbon dot structures could selectively stain Gram-positive over Gram-negative strains [51]. This same attention must be given to other Gram-types and to *specific* strains as well in the context of photodynamic carbon dots in APDT.

Further, there is a need for thorough characterization of the experimental parameters required to achieve bactericidal capabilities; that is, for what conditions and for what bacterial strains is sterilization possible? Under what conditions is no effect observed for the same strain? This thorough reporting is absolutely essential, because as with small molecule photosensitizers, the effectiveness of APDT from carbon dots is largely dictated by parameters such as localization in or near to cells, achieving adequate light doses, and optimizing the concentration ratio of bacterial cells to carbon dot sample [16]. This is particularly important in the context of bacterial resurgence, which is known to be a challenge in photosensitization technologies [3] and is of yet not frequently examined in carbon dot APDT studies. In fact, even for the current studies of initial antibacterial effects, some controls themselves may not be truly sufficient, as antimicrobial efficiency of different carbon dot structures are compared based largely on a mass-dependent concentration of carbon dots (for example, N μg/mL); this does not necessarily take into account the differences in size, elemental composition, or even photophysical differences such as the nanoparticle extinction. One potential strategy to probe this effect is by re-framing concentration comparisons in terms of carbon dot absorption values at the excitation wavelength in question, as we have done recently [56]. By this method, the efficiency of ROS generation by photosensitization mechanisms specifically may be examined for different structures. Even in this regime, though, considerations such as localization count of carbon dots at or near bacterial surfaces should be accounted for in additional experiments, along with analysis of any dark toxicity mechanisms that may also be at play. All of these considerations and more have begun to be addressed via studies focused on optimizing the antibacterial response, as discussed in the following section.

### 3.3. Optimization of Antimicrobial Activity: Tuning of Luminescence Quantum Yields, Precursor Selection, Surface Charge, and Experimental Parameters

**Figure 6:**
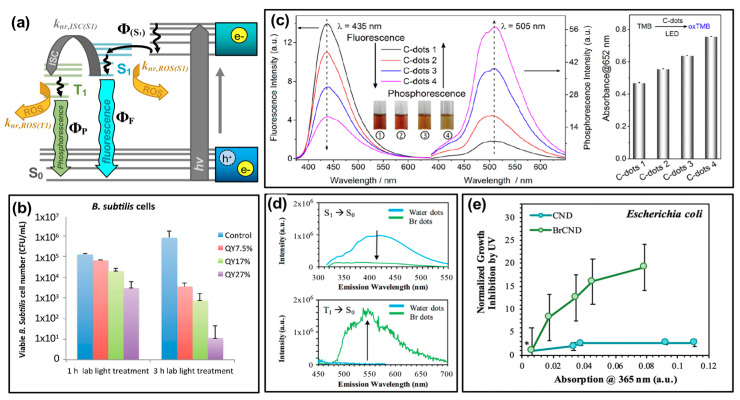
Tuning the quantum yield (Φ) values of carbon nanodots for optimized antimicrobial activity. (**a**) Modified Jablonski diagram showing initial absorption by carbon nanodots and subsequent transition to an emissive excited state (Φ_S1_), whereby the state may radiatively relax (Φ_F_) or undergo non-radiative transitions including intersystem crossing (“ISC”, k_nr,ISC(S1)_) to an excited triplet state (T_1_) or generation of reactive oxygen species (ROS). (**b**) Viability of *Bacillus subtilis* after treatment with light-activated carbon nanodots of varying fluorescent quantum yield, “QY”. (**c**) *Left*—Fluorescence and phosphorescence emission spectra for comparable carbon nanodot structures displaying variable quantum yield values (C-dots 1–4). *Right*—Detection of reactive oxygen species by TMB (3,3′,5,5′-tetramethylbenzidine) absorption increases, correlating to the phosphorescence quantum yield of varying carbon dot species. (**d**) *Top*—Fluorescence decrease from carbon dot samples (CND/“water dots”) following bromination (BrCND/“Br dots”) and *bottom*—corresponding changes in phosphorescence for the same samples. (**e**) Growth inhibition of *Escherichia coli* for phosphorescent carbon dots (BrCND) versus fluorescent carbon dots (CND) of varying concentrations (reported by corresponding absorption values); values were normalized to those for a non-BrCND-containing solution (*), from Reference [56]. For additional details regarding the reported studies, the reader is referred to Table 1 of the main text. **Abbreviations:** k_nr,ISC(S1)_ (rate of ISC from S_1_ excited state), k_nr,ROS(T1)_ (rate of ROS generation from triplet states), k_nr,ROS(S1)_ (rate of ROS generation from singlet states), oxTMB (oxidized TMB). **Permissions**: (**b**) Adapted from Reference [24]—Published by The Royal Society of Chemistry. (**c**) Adapted with permission from Reference [55]. Copyright © 2018, American Chemical Society. (**d**) Adapted from Reference [46]—Published by the PCCP Owner Societies.

A number of strategies have been introduced recently to tune the antimicrobial efficiency of carbon dot photosensitizers. Among these, optimization of photophysical properties—namely, quantum yields—has emerged as a promising avenue for achieving more effective structures. In the discussion of quantum yield values regarding photosensitizers, it is important to consider both fluorescence and phosphorescence as ROS formation may occur competitive to either emissive pathway, as illustrated in Figure 6a. For fluorescence, let us first consider a simple case (“Case 1”), where radiative pathways are depicted as an excitation event to a vibronic level of the S_1_ state, followed by non-radiative relaxation and ultimately decay in the form of fluorescence, as illustrated in previous figures. This process has a quantum yield of Φ_F,1_, determined by the ratio between the radiative (k_r,(S1)_) and non-radiative (k_nr,x(S1)_) decay routes of S_1_ “x(S_1_)”, shown in Equation (1):

(1) $$\Phi_{F,1}\;=\;\frac{k_{r,\left(S1\right)}}{k_{r,\left(S1\right)}+\sum^{\;}k_{\mathrm{nr},\;x\left(S1\right)}}\;,$$

where ROS formation from the S_1_ excited state (k_nr,ROS(S1)_) then is considered one of the non-radiative decay pathways included in the sum. Since quantum yield considers that all excitation events result in some form of deactivation, the overall magnitude of ROS generation and emission for a given period of excitation events, therefore, increases proportionally to excitation, as all excitation events in this simplified regime result in S_1_ formation.

This description hinges on two key assumptions: (1) It is the emissive, S_1_ energy and electron configuration that is responsible for ROS generation from carbon dots, and (2) carbon dots fluorescence and generate ROS solely from S_1_ and no other S_n_ state. This is indeed simplified, as carbon dots have been shown to exhibit excitation-dependent emissive behavior. Nonetheless, the “Case 1” description of fluorescence quantum yield gives valuable insight into the interplay between radiative emission processes and ROS formation for these structures and lends merit to their study and discussion for APDT optimization of carbon dots. Under the aforementioned assumptions, a higher fluorescence quantum yield photosensitizer would be disadvantageous, demonstrating a higher probability for radiative decay versus deactivation by formation or reaction of ROS (non-radiative). In carbon dots, as mentioned, it is unlikely though that excitation would occur directly to the S_1_ state, and in fact, may initially have non-emissive character upon e^−^/h^+^ formation. This more complex regime will be considered “Case 2”. The simplified Jablonski diagram of Figure 6a has been modified accordingly to include a non-emissive nth excited state; through non-radiative pathways, this state relaxes to S_1_, forming the emissive excited configuration, with a corresponding quantum yield of formation noted by Φ_(*S*1)_. It is important to note here, however, that a key assumption is still at play: that the S_1_ state is solely responsible for ROS generation in carbon dot systems. This “Case 2” model does not encompass the likelihood that additional emissive or non-emissive excited states may undergo Type I or Type II photosensitization mechanisms to produce ROS. Nonetheless, the following description can illuminate seemingly counterintuitive correlations between fluorescence quantum yields and antibacterial effects from ROS reported by some authors [24]. Returning to the discussion of S_1_ formation, the quantum yield equation can subsequently be adapted to reflect the non-radiative processes involved in emissive state formation, as in Equation (2):

(2) $$\Phi_{\left(S1\right)}\;=\;\frac{k_{\mathrm{nr},\;\mathrm{Sn}\to S1}}{k_{\mathrm{nr},\;\mathrm{Sn}\to S1}+\sum^{\;}k_{\mathrm{nr},\;x\left(\mathrm{Sn}\right)}},$$

where k_nr,Sn→S1_ reflects the cumulative decay rate of the excited state S_n_ to S_1_, and Σk_nr,x(Sn)_ is the sum of all other non-radiative decay rates from the S_n_ excited state. It should be noted here that while we have designated the formation of S_1_ as a variable of quantum yield (Φ), all rates discussed in equation 2 are for non-radiative processes. In this instance, what is therefore key is that an excitation event to an S_n_ excited state introduces a multitude of additional non-radiative pathways that will decrease the simplistic, “Case 1” fluorescence quantum yield (Φ_F,1_) by the factor Φ_(S1)_ as shown by Equation (3):

Φ_F,2_ = Φ_F,1_Φ_(S1)_, (3)

This concept was discussed by Al Awak et al. in the context of their results (Figure 6b) [24], although the photophysical and mathematical examination of these complex properties included herein is our own. In the consideration of “Case 2”, raising the overall fluorescence quantum yield (Φ_F,2_) could either reflect an increase in radiative efficiency from S_1_ (that is, an improvement in Φ_F,1_) and/or an increase in Φ_(S1)_. In the former case, this improvement would not lead to higher ROS yields, as radiative relaxation is a process that runs competitive to non-radiative ROS formation (k_nr,ROS(S1)_). When the quantum yield improvement comes instead by optimizing the formation of the S_1_ state (that is, Φ_(S1)_) this leads instead to an overall higher population of emissive states capable of forming ROS competitively to the radiative relaxation reflected by Φ_F,1_. The expected result, in this case, would be that a higher Φ_F,2_ could lead to more pronounced antibacterial activity. In fact, this is what Al Awak et al. reported, as shown in Figure 6b using *Bacillus subtilis* (*B. subtilis*) cells. By separating EDA-CDots into three discrete fractions using an aqueous gel column, the authors obtained samples that exhibited only minor spectral profile variations in absorption and fluorescence, but significantly different detected quantum yields (Φ_F_ = 7.5–25%). The authors also confirmed the higher antibacterial efficacy of the high quantum yield sample with *E. coli* cells, although the overall response was less pronounced than that reported for *B. subtilis* [24]. In another study, by Jijie et al., a decrease in fluorescence quantum yield upon ampicillin functionalization of carbon dots correlated also to a decrease in singlet oxygen formation [17]. Although fluorescence quantum yield tuning was not the focus of the publication, this observation provides further support for the correlation. It is interesting to note, however, that the relative influence of fluorescence quantum yield on antibacterial effects is also largely dependent on other factors as well, including carbon dot surface charge. In a study by Abu Rabe et al., carbon dots passivated by oligomeric polyethylenimine (PEI) were compared to those prepared by carbonization of a PEI/citric acid mixture [21]. For these particles, reported quantum yields were ~12% and ~60% respectively; however, the PEI-functionalized particles were positively charged at ~neutral pH, while the PEI/CA dots exhibited zwitterionic pairs that approximated a neutral charge under the same conditions. The authors reported that despite the quantum yield differences, the antibacterial effect on *B. subtilis* cells was dominated rather by the surface charge effects. This phenomenon has been further studied and will be the focus in a later section.

In the discussion of quantum yield regarding ROS formation and antibacterial efficacy, it is important as well to address phosphorescence pathways, which permit the formation of singlet oxygen. For highly phosphorescent agents, intersystem crossing (k_nr,ISC(S1)_) is favorable (Figure 6a); as such, corresponding fluorescence intensities and therefore quantum yields may be low due to competitive decay from triplet excited states, expanding Equation (1) to Equation (4):

(4) $$\Phi_{F,1}\;=\;\frac{k_{r,\left(S1\right)}}{k_{r,\left(S1\right)}+\sum^{\;}k_{\mathrm{nr},\;x\left(S1\right)}+k_{\mathrm{nr},\mathrm{ISC}\left(S1\right)}},$$

The phosphorescence quantum yield once the T_1_ state is formed (Φ_P,1_) can then be considered as analogous to Equation (1) as well, shown in Equation (5):

(5) $$\Phi_{P,1}\;=\;\frac{k_{r,\left(T1\right)}}{k_{r,\left(T1\right)}+\sum^{\;}k_{\mathrm{nr},\;x\left(T1\right)}},$$

where k_r,(T1)_ is the radiative decay rate of phosphorescence from the T_1_ excited state, and *Σk_nr,x(T1)_* the corresponding summation of non-radiative decay processes, including ROS generation. Yet this description neglects the key factor of k_nr,ISC(S1)_; a more encompassing representation of phosphorescence quantum yield (Φ_P,2_) then may be reflected by Equations (6) and (7), analogous to Equations (2) and (3) respectively:

(6) $$\Phi_{\mathrm{ISC}}\;=\;\frac{k_{\mathrm{nr},\;\mathrm{ISC}\left(S1\right)}}{k_{\mathrm{nr},\;\mathrm{ISC}\left(S1\right)}+k_{r,\left(S1\right)}+\sum^{\;}k_{\mathrm{nr},\;x\left(S1\right)}},$$

Φ_P,2_ = Φ_ISC_Φ_P,1_ (7)

where the “quantum yield” of ISC to form the excited (emissive) triplet state is given by Φ_ISC_. It becomes obvious then that phosphorescence quantum yield may be considered for antibacterial applications in a similar way to “Case 2” fluorescence quantum yield discussed earlier. By improving Φ_ISC_ of a species, a more excited triplet character can be achieved. Given the unique triplet-triplet interaction possible between these excited states and ground-state oxygen, optimization of triplet character from carbon dots is a natural avenue to improve antibacterial efficiency. The phosphorescent quantum yield is an effective measurement to probe triplet character in a photosensitizer; this is particularly true as phosphorescence detection typically requires fixation in a matrix [55] or minimization of oxygen diffusion [46] since oxygen so readily quenches radiative decay from triplet excited states. This necessitates essentially the removal of k_nr,ROS(T1)_ as a possible decay pathway such that phosphorescence can be observed. It can then be assumed that a photosensitizer displaying a strong phosphorescent signal will also likely interact strongly with oxygen to form ROS, since (for detection) k_nr,ROS(T1)_ is mitigated. This has in fact been confirmed by both Zhang et al. [55] and by us [56], using different synthetic avenues to achieve phosphorescent carbon dot structures. Work by the former is displayed in Figure 6c; using hydrothermal polymerization of citric acid and ethylenediamine, the authors obtained carbon dot samples of differing phosphorescence quantum yields. As shown by the luminescence spectra, as fluorescence intensity decreases for these samples, phosphorescence intensity inversely increases, consistent with the photophysical mechanism of phosphorescence. To probe the generation of singlet oxygen, the authors monitored the colorimetric probe 3,3′,5,5′-tetramethylbenzidine (TMB), which upon oxidation by singlet oxygen increases in absorption at 652 nm. The results of this study are presented in Figure 6c (right). It is clear that the degree of TMB oxidation, and therefore singlet oxygen production, correlates also to the carbon dot phosphorescence quantum yield. The authors indicate that the overall nitrogen content in their carbon dot structures is likely the source of the phosphorescence differences, and therefore presents a strategy for phosphorescence optimization in other carbon dot syntheses [55,69].

They do additionally include analysis of phosphorescent “graphene quantum dots” as well, in which sulfur-doped structures (labeled “GQD2”) were compared to the un-doped “GQD1”. With sulfur doping, the “heavy atom effect” was employed to improve the favorability of intersystem crossing, thereby achieving a more phosphorescent and subsequently stronger antibacterial photosensitizing agent comparatively [55]. The heavy atom effect may also be employed by the incorporation of other atoms such as bromine or iodine [70]. Recently we have shown that intersystem crossing, and subsequently phosphorescence, may also be optimized in carbon dots via bromination (Figure 6d) or, less effectively based on synthetic limitations, iodination [46]. As shown in Figure 6d, a control of fluorescent “water dots”—that is, carbon dots collected from methane combustion into deionized water, in the absence of bromine—were compared to the condition were carbon dots were brominated during collection, labeled “Br dots”, referred to later as “BrCND”. Upon bromination, the fluorescence intensity of water dots diminishes, while a new phosphorescent peak appears at approximately 550 nm. Under comparable conditions, viability studies were conducted using *E. coli* for both bromine dots (“Br dots”, BrCND) and carbon dots (“water dots”, CND), as shown in Figure 6e [56]. Absorption values were recorded for carbon dot samples at different dilutions and plotted against the antimicrobial photodynamic factor from UV photosensitization (the process of analysis was identical to that reported in Figure 4a,b). Samples were normalized to a buffer-only exposure solution (*) containing no carbon dot structures. It is interesting to note that no significant photodynamic effect is observed for the fluorescent CND sample; the phosphorescent brominated structure, however, exhibits a significant photodynamic response that is also concentration-dependent. Further, a study by Mandal et al. observed a decrease in fluorescence quantum yield following BSA conjugation to carbon dots; this decrease also correlated to increase ROS production [3]. Although phosphorescence itself was not detected, the authors identified the mechanism of ISC as a potential explanation for this observation. From these results and those discussed previously, it becomes clear that phosphorescence tuning is a competitive potential strategy for optimizing the antimicrobial response from carbon dot photosensitizers in future research.

In the previously noted Zhang et al. study, the authors generated their phosphorescent structures by altering the amount of ethylenediamine precursor in synthesis to tune final nitrogen concentration [55]; in our work, a bromine component was added as a precursor [46,56]. In fact, precursor tuning has been a mainstay strategy in carbon dot literature to achieve new and varied properties for a multitude of applications, and APDT is no exception. In the vein of phosphorescence discussion, Mandal et al. selected an anthraquinone derivative known as Anthrarufin for their carbon dot precursor specifically due to its known triplet character [3]. Beyond photophysical tuning, precursor selection has prioritized retaining antimicrobial properties [51,71,72] or even cell targeting capabilities [19] by using antibacterial small molecule drugs as precursors. In a general sense, precursors may also be selected simply to improve the stability of and reduce defects in the resulting carbon dots after carbonization, for example in the study by Kavitha et al. which used date palm fronds (featuring a high lignin content) as a biomass precursor [49]. Although many studies cite the rationale for precursor selection, comprehensive and methodical examinations of precursor variations and their downstream influence on carbon dot properties are limited. Nonetheless, this technique will likely receive expanded attention in regard to the development of new photodynamic antimicrobial carbon dots.

**Figure 7:**
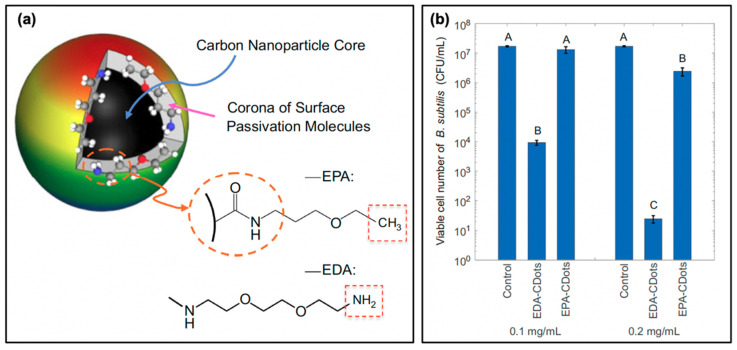
Surface charge effects on antimicrobial activity. (**a**) Schematic showing the surface passivation agents used to tune the surface charge of carbon nanodots, whereby 2,2-(ethylenedioxy)bis-(ethylamine) (EDA) confers a positive charge and 3-ethoxypropylamine (EPA) a neutral charge at pH 7.4. (**b**) Viability of *Bacillus subtilis* cells following photodynamic treatment with EDA- and EPA-conjugated carbon dots (EDA/EPA-CDots). The control consists of the light-exposed samples in the absence of carbon dot treatment. Statistical difference is indicated by the letters (A–C, *p* < 0.05); data with the same letter label have no statistical difference. For additional details regarding the reported studies, the reader is referred to Table 1 of the main text. **Permissions**: (**a**,**b**) Adapted from Reference [21]. Open access.

Another consideration in the selection of precursors or passivation agents is one that has been previously mentioned: carbon dot surface charge. Already it has been noted that the carbon dot charge has a notable impact; these effects have been studied by Abu Rabe et al. as mentioned in the discussion of quantum yield effects; an additional snapshot of their results is provided in Figure 7 [21]. The authors first prepared the carbon dots, followed by a functionalization procedure with either 2,2-(ethylenedioxy)bis-(ethylamine) to produced EDA-CDots, or with 3-ethoxypropylamine to form EPA-CDots. A simplified schematic and molecular structures are provided in Figure 7a. It should be noted that both structures exhibited diameters of ~4–5 nm, with fluorescence quantum yields of ~20%; however, at neutral pH, the EPA substituents carry no charge. Conversely, EDA will be protonated at this pH, and therefore carry a positive overall charge. The authors subsequently conducted bacterial viability studies at approximately neutral pH using *B. subtilis* cells, the results for which are given in Figure 7b for two different concentrations of carbon dots. From these data, it is clear that the antibacterial activity of the EDA-CDots significantly exceeds that from the EPA-CDots; this clearly illustrates how the positive charge on EDA-CDots could assist in the electrostatic attraction of the carbon dots to negatively charged bacterial membranes. Although this localization was not specifically probed by the authors, it seems plausible that such attractive forces would permit localization of the carbon dots, improving the bactericidal efficiency of generated ROS.

The authors also tested this using PEI/CA-CDots synthesized with varying PEI:CA (and therefore –NH_2_ vs. R–NH_2_^+^CA^−^) ratios, thereby tuning the pKa while size (~10 nm) and quantum yield (~60%) stayed constant between samples. Similar to the results for Figure 7, the authors found an increase of bactericidal efficiency correlating to the degree of protonation at near-neutral pH and therefore surface charge. This effect was found to be further pronounced when using PEI-coated carbon dots with a smaller molecular weight PEI passivation agent (PEI_600_ versus PEI_1200_), perhaps due to the decreased distance of ROS diffusion, although this mechanism was not studied explicitly in the report [21]. Although this is promising, more extensive characterization is required to truly understand the effects that surface charge can have during carbon dot photocatalytic disinfection or APDT. For example, in one study it was found that carbon dots carrying a negative zeta potential were unable to stain live cells, consistent with the trends discussed previously; however, photodynamic results for these carbon dots were still observed [28]. Still another study reported that carbon dots with negative zeta potentials were effective against Gram-positive bacteria, and in fact underwent cellular uptake [51]. These authors suggested that the “porous” nature of Gram-positive membranes permitted the carbon dots to permeate into the cell; subsequently, damage could be inflicted leading to cytoplasmic membrane disruption, alterations of cell permeability, and even leakage of intracellular components. In fact, several reports have indicated that negatively-charged carbon dots are effective against either Gram-positive or Gram-negative bacteria [17,49,56].

**Figure 8:**
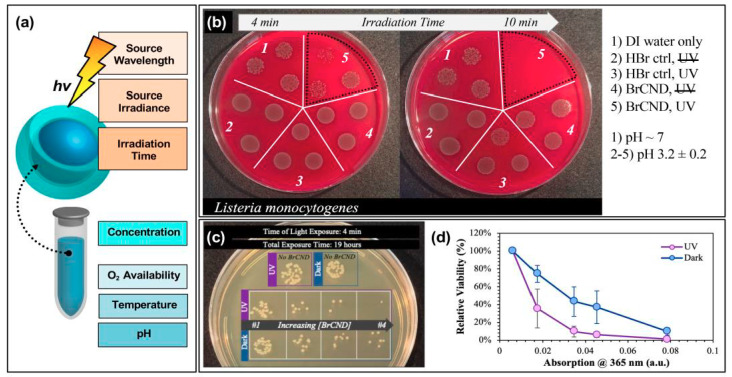
Parameters for tuning carbon nanodot photodynamic antimicrobial effects. (**a**) Schematic displaying some of the experimental parameters that must be adjusted to achieve maximal photodynamic antimicrobial effects from carbon nanodots. (**b**) Sample data set demonstrating the impact of irradiation time on the colony growth for *Listeria monocytogenes* exposed at 365 nm with brominated carbon nanodot (BrCND) photosensitizers. (**c**,**d**) Viability of *Escherichia coli* (**c**) grown on plates and (**d**) plotted as a function of photosensitizer concentration (plotted here by the BrCND sample absorption at 365 nm). Panels (**b**–**d**) adapted from Reference [56]. For additional details regarding the reported studies, the reader is referred to Table 1 of the main text.

One possible source of these seemingly conflicting results on carbon dot surface charge is the complex interplay between all of the factors influencing antimicrobial efficacy in APDT and photocatalytic disinfection. We have seen how variations in quantum yield and surface charge can elicit two very different antimicrobial responses, and logically the ratio of bacterial to carbon dot concentrations applied experimentally will be a large determinant on efficacy, including considerations such as bacterial resurgence. The immense variability in experimental parameters for antimicrobial photosensitization in general, let alone for carbon dots themselves, once again calls for methodical approaches to analyze these factors. A few key determinants for optimizing antibacterial responses simply by adjusting experimental parameters are given in Figure 8a.

These can be considered to fall into roughly two categories: variables of excitation or “solution” (in this case, used to refer to the experimental environment) conditions. For the former, aspects such as the excitation wavelength, the source irradiance or power, as well as the irradiation time will all influence the observed antibacterial response. Take for example the time-dependent results for *Listeria monocytogenes* growth following exposure with brominated carbon nanodots, shown in Figure 8b [56]. In this study, reported recently by our lab, some impairment of growth was observed for *Listeria monocytogenes* (*L. monocytogenes*) samples that underwent a 4-min exposure period with bromine dots (followed by plating and overnight incubation at physiological pH). In an *n* = 3 sample set, however, there were instances where growth in this regime closely matched that observed for the control samples. Using a 10-min exposure period, however, virtually no growth was observed for the irradiated BrCND sample. Looking only at the 10-min regime, one might expect the bromine dots to be highly efficient at killing *L. monocytogenes*; however, it cannot be neglected that incomplete inactivation occurs on shorter time scales; subsequently, this experimental parameter must be optimized on a case-by-case basis. Regarding the other excitation parameters for the same study, UV light sources proved more efficient in producing antibacterial responses under the same experimental constraints as compared to room light; this could be due to differences in irradiation doses, or in the photophysical response of the carbon dot to select wavelengths, or—more likely—a combination of the two [56]. Other studies have similarly reported significant influence on observed response from varied exposure times [17,19,24,25,26,28,49,55], excitation sources [24], and powers [17].

In addition, the “solution” parameters that affect antibacterial response include the carbon dot concentration, oxygen (or more generally, ROS precursor) availability, reaction temperature, and pH to name a few. In our report of bromine dots, for example, an antibacterial effect would likely not be observed, or would at least be observed to a lesser extent, if the exposure were to be conducted at neutral or basic pH; in these environments, there is less triplet character which subsequently should result in a lower concentration of singlet oxygen generated for APDT [46]. Oxygen dependence of singlet oxygen production from bromine dots was also reported [56]. Zhang et al. determined that higher temperatures yielded less photosensitization, clearly illustrating the need for temperature as a controlled parameter in these experiments [55]. Regarding carbon dot concentrations, numerous studies have highlighted the dependence of photosensitization effects on the “dose” of carbon dots [16,17,24,25,26,28,56]. One such instance as reported by our lab is illustrated in Figure 8c,d [56]. The growth of *E. coli*, both under dark and exposed conditions, is highly dependent on the concentration of bromine dots (dilutions represented by their corresponding absorption values at 365 nm, normalized at 100% viability for the “No BrCND” control solution absorption value). From these results, it is clear that not only must the carbon dot concentration be characterized carefully from the perspective of the overall detected photodynamic response, it also heavily influences the detection of any potential “dark toxicity” mechanism (Figure 8d). Considering all of these factors, it is even possible to “normalize” the antibacterial response between different carbon dot samples for comparison, as was reported by Al Awak et al. in 2017; in this study, the authors tuned both the exposure times and/or the dose concentrations of their varying fluorescence quantum yield fluorophores to achieve statistically comparable antibacterial responses in *B. subtilis* cells [24]. The antimicrobial properties of photo-responsive carbon dots are indeed encouraging for the future development of these particles as agents for APDT and photocatalytic disinfection; however, the existing chemical, physical, and photophysical underpinnings governing the efficiencies of carbon dots in this context remain to be methodically studied and elucidated.

## 4. Carbon Nanodots in Hybrid Photodynamic Systems

### 4.1. Systems Containing Carbon Nanodots with Intrinsic Antimicrobial Character: Key Studies and Considerations

**Figure 9:**
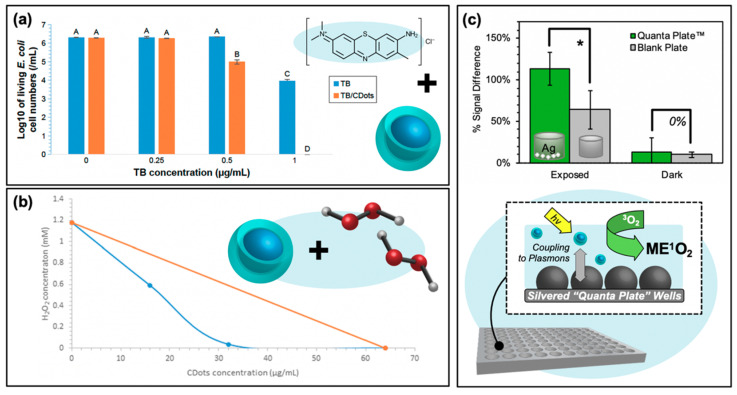
Carbon nanodots in hybrid systems for optimizing reactive oxygen species generation. (**a**) Synergistic decrease in *Escherichia coli* growth resulting from carbon nanodot co-photoactivation with the toluidine blue (TB) photosensitizer. Statistical difference is indicated by the letters (A–D, *p* < 0.05); data with the same letter label have no statistical difference. (**b**) Isobologram demonstrating the synergistic effects of hydrogen peroxide/photoactivated carbon dot co-administration in achieving complete bacterial inhibition. (**c**) *Top*—Metal-enhanced singlet oxygen generation (ME^1^O_2_) detected using fluorescence-on probe Singlet Oxygen Sensor Green in blank or silver nanoparticle-coated “Quanta Plate™” wells; * *p* < 0.10 (from Reference [60]). *Bottom*—Schematic of ME^1^O_2_ by light-activated carbon nanodots in proximity to nanoparticulate silver island films. For additional details regarding the reported studies, the reader is referred to Table 2 of the main text. **Permissions**: (**a**) Adapted from Reference [16]. (**b**) Adapted from Reference [73].

Although the fundamental research surrounding the mechanisms and optimization of carbon dots as photosensitizers is still improving, researchers have already begun to explore how these particles may be incorporated into hybrid systems for even more efficient antibacterial properties. In some of these instances, the intrinsic antibacterial properties of the carbon dots themselves are employed to improve the overall system efficiency. Instances where the photocatalytic properties of the hybrid system are optimized are presented in Figure 9. One strategy presented by Dong et al. involves the synergistic capabilities of co-administered carbon dot concentrations with small-molecule photosensitizers methylene blue (MB) and toluidine blue (TB) [16]. The results for TB co-administered with 5 μg/mL of carbon dots are presented in Figure 9a (experimental details for figures in this section expanded in Table 2, shown previously). When carbon dots are administered alone ([TB] = 0 μg/mL), no appreciable antibacterial activity is observed (it should be noted briefly, however, that for carbon dots alone, antibacterial effects from photosensitization were observed for higher concentrations). As the concentration of co-administered TB is increased, however, clear synergistic activity was observed; a similar effect was observed for MB as well. Although “partial synergism” was reported for a variety of CDot:MB concentrations, the one condition for which the authors indicated synergy was 40 and 8 μg/mL of carbon dots and methylene blue respectively. This compares to the minimum inhibitory concentrations (MIC) of carbon dots at 160 μg/mL and methylene blue at 32 μg/mL. Although not explicitly studied, a number of reasons for this observed synergism are reported by the authors, including (1) that the small molecule photosensitizer increases cell permeation, (2) that carbon dots improve the solubilities of their small-molecule counterparts, thereby improving uptake/localization, or (3) that the combination of both photosensitizing agents increases the overall intracellular ROS, for example by a fluorescence resonance energy transfer (FRET) mechanism. This final explanation seems likely, as the presence of carbon dots noticeably increased the ROS detection relative to MB alone; it was similarly noted that the carbon dots produced little ROS themselves at the concentration used. The authors go on to discuss the possibility that carbon dot emission can instead excite photosensitizers rather than emitting, thereby increasing the overall absorption of the system [16]. This description, however, requires closer characterization to validate. In a report of another hybrid system, Dong et al. instead administered the carbon dot photosensitizers in conjunction with hydrogen peroxide [73]. Synergistic effects were also observed here, as shown by the isobologram in Figure 9b. While the MIC of photoactivated carbon dots against *E. coli* cells was 64 μg/mL and that of H_2_O_2_ 1.176 mM (orange series), combinations of the two species yielded comparable antibacterial activities with much lower concentrations of each agent (blue series). If one returns to Reaction (R 6) (H_2_O_2_ + e^−^ → OH^−^ + ^•^OH), a clear mechanism of this enhanced activity is evident. With a higher concentration of reagents available to react with the photo-generated e^−^/h^+^ pairs from carbon dots, a higher concentration of ROS is also possible [73].

Further, it may assist with biasing the formation of specific ROS from carbon dot photoactivation, although this is not discussed. Co-administration of carbon dots and hydrogen peroxide has also been performed by others, particularly for the photocatalytic degradation of organic dyes [49].

Another system for enhanced APDT is shown in Figure 9c. Rather than employing a co-administration strategy, this platform takes advantage of through-space fluorophore-plasmon interactions to amplify the generation of reactive oxygen species. This amplification platform has its mechanistic basis in metal-enhanced fluorescence (MEF), whereby a plasmonic metal substrate can act as a “nanoantennae” for excitation light; the resulting field generated by the substrate may then excite larger populations of fluorophores in the near-field than would be possible under classical conditions. This effectively expands the absorption cross-section well beyond what would be possible for the fluorophore alone and is known as the enhanced absorption mechanism. MEF may also occur as a result of enhanced emission pathways, where fluorophore quanta couple to the nanoparticle plasmons such that the whole entity radiates as a unit [75]. MEF, and even metal-enhanced phosphorescence (MEP), from carbon dots on silvered substrates have been previously reported by our lab [76,77]. However, the enhancement of radiative emission could actually run counter to the metal enhancement of ROS (ME-ROS), as radiative decay versus ROS generation are competitive decay pathways for excited carbon dots. Nonetheless, ME-ROS has been detected for small molecule photosensitizers, potentially via enhanced absorption, yielding ME^1^O_2_ [78,79] and MEO_2_^•−^ [78,80]. In a recent study by our lab, ME^1^O_2_ was also detected from carbon dots on silvered substrates, shown in Figure 9c [60]. This enhancement was detected using the fluorescence-on probe Singlet Oxygen Sensor Green™ and is reported as a function of percent signal difference, that is, the difference or increase between the final probe fluorescence intensity after photosensitization and its initial intensity. This analysis was conducted for photosensitization exposure conditions contained both in the silvered wells or in a blank well plate as a classical control. Fluorescence detection for all samples was performed in a blank 96-well plate. The results demonstrate a greater signal increase for the silvered exposed wells than detected for the blank plate; both plates yielded statistically similar “dark” results, confirming ME^1^O_2_. This technology remains to be further refined and tuned, with a vast array of metal substrates available to optimize the enhanced absorption rather than emission coupling mechanism for carbon dots in ME-ROS systems; however, these results do in fact reveal an additional way that carbon dots may be employed in APDT hybrid systems. This application of a carbon dot/silver nanoparticle hybrid is particularly intriguing, as typically these capitalize primarily on the inherent antibiotic character of the silver particles [81,82], employing carbon dots instead as either imaging agents [83] or as reducing agents in the initial silver particle synthesis [84,85,86,87,88].

**Figure 10:**
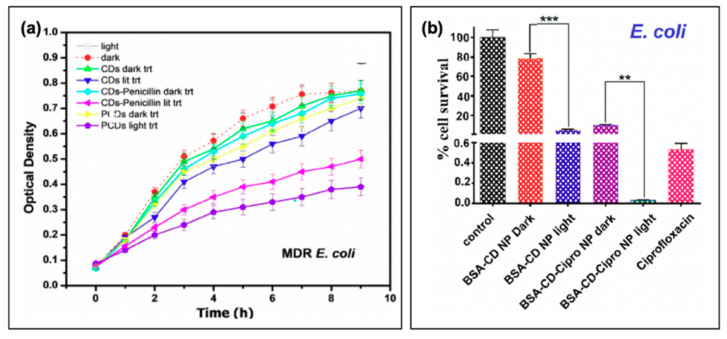
Carbon nanodots in hybrid systems with antibiotics. (**a**) Bacterial growth curves for multi-drug resistant (MDR) *E. coli* with treatment using either penicillin conjugated (CDs-Penicillin) or penicillin precursor (PCDs) carbon dots (CDs). (**b**) Cell viability of *Esherichia coli* after treatment with the carbon nanodot structure (BSA-CD NP) alone or loaded with ciprofloxacin (BSA-CD-Cipro NP) both under light and dark conditions (**, 0.05 < *p* < 0.01; ***, 0.01 < *p* < 0.001). For additional details regarding the reported studies, the reader is referred to Table 2 of the main text. **Abbreviations:** BSA (bovine serum albumin), NP (nanoparticle), CD (carbon nanodot). **Permissions**: (**a**) Adapted with permission from Reference [19]. Copyright © 2017, John Wiley and Sons. (**b**) Adapted with permission from Reference [3]. Copyright © 2019, American Chemical Society.

Carbon dot photosensitizers have also been incorporated into antibiotic-containing regimes, such that complementary mechanisms of antibiotic action and oxidative damage act to stop bacterial growth. An example of a study by Sidhu et al. is shown in Figure 10a, in which the authors used penicillin as both a carbon dot precursor (PCDs) or passivation agent (CDs-Penicillin) [19]. As evidenced by the growth curves of multi-drug resistant (MDR) *E. coli*, CDs alone had only a limited impact on bacterial growth, and only under photosensitization conditions. Incorporation of penicillin, in both regimes, led to repressed observed growth under dark conditions. This effect was further amplified by light exposure, confirming the complementary mechanisms from antibiotic properties and photosensitization [19]. Another study that was briefly mentioned previously was performed by Jijie et al., in which ampicillin (AMP) was functionalized to carbon dot structures after their synthesis [17]. It was found by SEM that AMP significantly enhanced the toxic “dark” effects observed for CD-AMP versus CDs alone; the activity was also improved compared to free AMP. The authors attributed this potentially to the ability of the CD structures to localize AMP at the bacterial membrane, such that the cell wall was exposed to a higher density of antibiotics. Under photosensitization conditions, reduction of viability was further enhanced for the CD-AMP structures, and bactericidal effects were possible with much lower concentrations. As an additional bonus, it was found that the long term stability of AMP was in fact also improved by functionalization to carbon dots [17]. Beyond simple passivation with antibiotics, researchers have also begun to explore the drug-loading capabilities of carbon dot complexes.

In a study by Mandal et al., BSA-functionalized carbon dot nanoparticles (~350 nm in size, from ~5 nm carbon dots) were subsequently loaded with ciprofloxacin; the results of this are presented in Figure 10b for *E. coli* [3]. Inclusion of the drug was found to improve the dark toxicity, similar to the other discussed reports of antibiotic co-administration, with a slow release of the ciprofloxacin (~80% release after 24 h). With light exposure, further growth reduction was once again observed, in this case for both *E. coli* and *S. aureus*, although the survival of *S. aureus* (0.47%) was more pronounced than that of *E. coli* (0.03%); this is to a degree to be expected, however, as ciprofloxacin is more effective against *E. coli* than *S. aureus* [3]. Carbon dots have also been incorporated with chitosan for wound healing in rats [89], and as ciprofloxacin-conjugated drug carriers [90]. While these studies also present an excellent foundation for further development, studies into resistant development under these regimes and bacterial resurgence patterns will be beneficial as the field develops.

**Figure 11:**
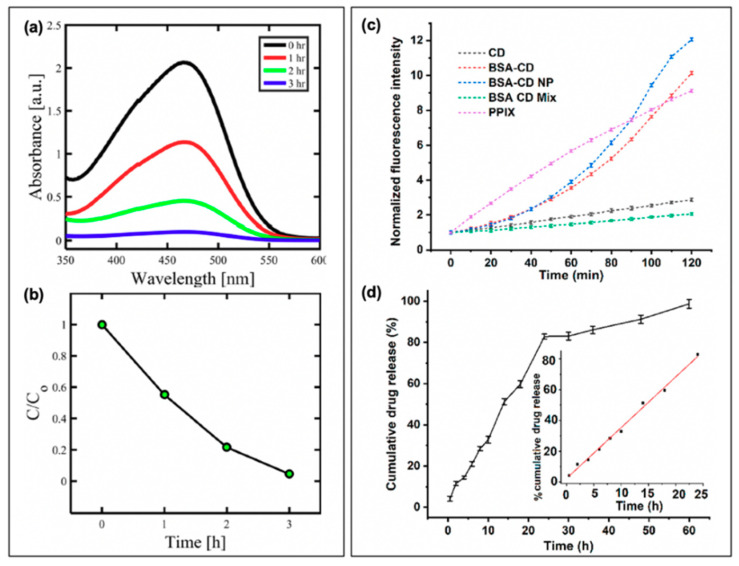
Considerations for maximizing the antibacterial effects for co-administration of carbon nanodots with small molecules. Examples include (**a**,**b**) dyes and (**c**,**d**) antibiotics. (**a**) Absorption spectra of methyl orange (MO) dye after irradiation of carbon nanodot treated solutions containing H_2_O_2_. (**b**) Photodegradation of MO dye. (**c**) Time-dependent reactive oxygen species (ROS) generation, detected by fluorescence-on probe Dihydrorhodamine 123. ROS generation is reported from carbon nanodots (CD), BSA-functionalized carbon nanodots (BSA-CD), nanoparticle structures (BSA-CD NP), BSA mixed with CDs (BSA CD mix), and a control photosensitizer, protoporphyrin IX (PpIX). (**d**) Time-dependent in vitro drug release of ciprofloxacin from BSA-CD NPs. For additional details regarding the reported studies, the reader is referred to Table 2 of the main text. **Abbreviations:** BSA (bovine serum albumin), NP (nanoparticle). **Permissions**: (**a**,**b**) Adapted from Reference [49], open access (licensed under Creative Commons Attribution 4.0 International License: http://creativecommons.org/licenses/by/4.0/). (**c**,**d**) Adapted with permission from Reference [3]. Copyright © 2019, American Chemical Society.

In hybrid systems, added complexity further necessitates careful consideration of potential undesirable interplay between the carbon dot photosensitization effects and whatever agent(s) accompany it in the system. For example, ROS from carbon dots have been reported to cause degradation of dyes as mentioned previously [22,49,59]. An example of this observation is shown in Figure 11a,b, in which methyl orange (MO) is significantly degraded by photoactivated carbon dots [49]. It should be noted that in this case, the carbon dots were also administered with hydrogen peroxide with the *aim* of photocatalytic degradation of the dye; however, the oxidative effects from even carbon dots alone could have severe deleterious effects on organic small molecules that have been incorporated as an essential part of the system. This interaction will be highly variable from system to system, dependent upon the photosensitizing characteristics of the employed carbon dots and the small molecule of interest and should be characterized in emerging studies of this nature. Another practical consideration for hybrid systems regards optimizing the timescale for drug release; as shown in Figure 11c,d for the ciprofloxacin-loaded BSA-CD nanoparticles, the timescales of ROS generation and drug release are not particularly comparable [3]. Whereas ROS generation was monitored on the scale of 0 to 120 min, 80% ciprofloxacin release was only achieved by 24 h. The measurement interval of the former was ~10 min, the latter was measured approximately every 2 h. It should come as no surprise that when combining drugs and photosensitization, the order in which antibacterial mechanisms will begin to act upon the cells must be considered. While this is in itself a challenge, it also provides an opportunity to understand how first weakening cells via one mechanism or the other could potentially improve the response of the secondary mechanism. Conversely, it may be found through future investigation that in some cases, simultaneous mechanistic timing is required. Nonetheless, administration timelines must be considered and tuned when optimizing carbon dot-based antibacterial dual-mechanism hybrid systems.

### 4.2. Systems Employing Carbon Nanodots to Improve Photophysical Properties: Fluorescence Resonance Energy Transfer and Upconversion Luminescence

**Figure 12:**
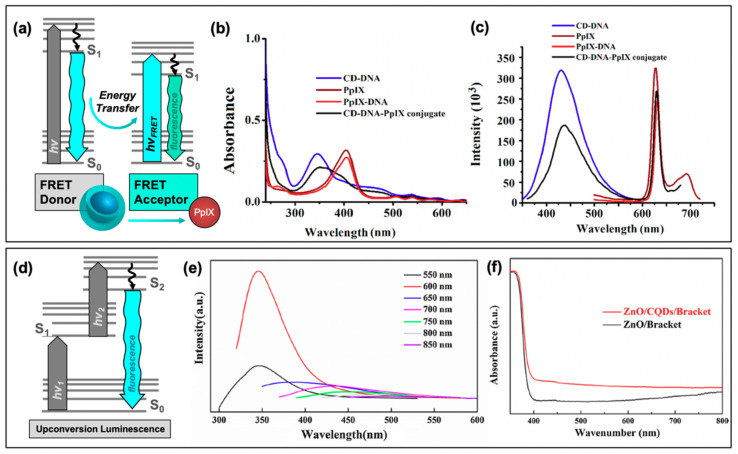
Carbon nanodots in photodynamic hybrid systems, including (**a**–**c**) fluorescence resonance energy transfer (FRET) and (**d**–**f**) up-conversion luminescence. (**a**) Modified Jablonski diagram demonstrating FRET between a donor (carbon dots) and acceptor (example: protoporphyrin IX, PpIX) molecule. (**b**) Absorption and (**c**) fluorescence emission spectra of DNA-functionalized carbon dots (CD-DNA), protoporphyrin IX (PpIX-DNA), and the hybridized form (CD-DNA-PpIX conjugate). These display the spectral overlap between FRET donor (CD-DNA) fluorescence and acceptor (PpIX) absorption. (**d**) Modified Jablonski diagram demonstrating a basic up-conversion process from two excitation events. (**e**) Excitation-dependent up-conversion fluorescence from carbon nanodots (CQDs) at varying excitation wavelengths. (**f**) Absorption increase of hybrid system (ZnO/CQDs/Bracket) relative to control (ZnO/Bracket). For additional details regarding the reported studies, the reader is referred to Table 2 of the main text. **Permissions***:* (**b**,**c**) Reprinted from Journal of Colloid and Interface Science, 533, Kumari, S. et al., “Carbon dot-DNA-protoporphyrin hybrid hydrogel for sustained photoinduced antimicrobial activity”, 228–238, Copyright © 2020, with permission from Elsevier [59]. (**e**,**f**) Reprinted from Chemical Physics Letters, 706, Zhang, J. et al., “Enhanced antibacterial properties of the bracket under natural light via decoration with ZnO/carbon quantum dots composite coating”, 702–707, Copyright © 2018, with permission from Elsevier [40].

While APDT effects have been reported from carbon dots in a handful of recently emerging studies, their other properties, namely photophysical properties, have conferred advantages for incorporation in photoactivatable antibacterial systems even when no intrinsic APDT characteristics are detected for the carbon dots alone. Studies have capitalized on their visible light absorption characteristics [40], broad-spectrum absorption [59], excitation-tunable fluorescence [91], and up-conversion luminescence [12] properties to name a few. Carbon dots have, for example, been employed as FRET donor agents for varied applications. A study by Kumari et al. used carbon dots in this capacity, designing a FRET-based hydrogel with protoporphyrin IX (PpIX) as the FRET acceptor and photosensitizing agent [59]. A simplified Jablonski diagram for this regime is provided in Figure 12a, whereby carbon dot emission energy is transferred to PpIX rather than emitting as fluorescence. To form the hydrogel, DNA linkers were used to create a system of carbon dots and PpIX; absorption spectra are provided in Figure 12b for the components, controls, and conjugate. As shown, PpIX primarily absorbs visible wavelengths, whereas the carbon dot component is a stronger UV absorber; accordingly, the authors used primarily UV excitation in their studies of ROS generation. As indicated by the fluorescence spectra in Figure 12c, UV excitation of the carbon dots results in an emission maximum of ~430 nm; this corresponds fairly well to the absorption peak of the PpIX component, resulting in emission detectable from both the carbon dot and PpIX components in the hybrid system. Unsurprisingly, when the hydrogel and controls were applied in bacterial viability studies, UV excitation of PpIX alone did not result in significant reduction of viability for *S. aureus* but did demonstrate efficacy with visible light excitation. Conversely, the conjugate hydrogel was effective under both UV and visible light activation. The carbon dot hydrogel alone had no detectable antibacterial effects. The authors found that they were able to mitigate all antibacterial activity by incorporating a ROS scavenger, confirming the antibacterial photosensitization mechanism. Interestingly, no antibacterial effects were observed across the board for *E. coli* samples. The authors indicate that this is likely due to the nature of Gram-negative membranes, which do not easily permit neutral protoporphyrins to permeate [59]. It is interesting to note that the authors chose to employ carbon dots in this instance for increased *short-wavelength* absorption. While UV activation is not typically desirable in APDT, it nonetheless is a component of sunlight. Therefore, in the goal of solar-driven photocatalytic systems, absorption of both UV and visible light to power antibacterial action is desirable.

The absorption of carbon dots has also been employed in hybrid systems in a different context; a simplified schematic for up-conversion luminescence is presented in Figure 12d. In this regime, the absorption of two lower energy wavelengths may result in emission from a higher energy state. This pathway is responsible for the observation of, for example, ~350 nm light from 550 nm excitation, as depicted (amongst other excitation energies) in Figure 12e [40]. In this study by Zhang et al., the authors employed carbon dots to convert visible light excitation to UV wavelengths, which the zinc oxide component of the overall hybrid material (ZnO/CQDs/Bracket) requires for activation. As evidenced in Figure 12f, incorporation of the carbon “quantum” dots (CQDs) increased the overall visible light absorption of the material as compared to the ZnO/Bracket structure alone (“bracket” refers to the material scaffold, which will be discussed further in the following section). Activation of the material by natural light, rather than carcinogenic UV exposure, was achieved [40]. In other instances, carbon dots have also been used to improve the visible light absorption characteristics of the hybrid. One previously mentioned study (Section 3.1) incorporated carbon dots to form a CDs/Na_2_W_4_O_13_/WO_3_ nanocomposite [12]. Upon incorporation, carbon dots lowered the fluorescence intensity of the system, possibly due to stabilizing e^−^/h^+^ formation in the material and preventing radiative recombination. This photoluminescence decrease corresponded also to increased photodisinfection efficiency with visible light irradiation of *E. coli* samples. Simultaneously, incorporation of carbon dots also broadened the absorbing region of the material [12]. In a report by Hazarika et al., carbon dots were used for both down- and up-conversion photoluminescence characteristics in a carbon dot and titanium dioxide (CD@TiO_2_) polyester nanocomposite material [27]. It is also worth noting that in some cases, carbon dots are simply incorporated for their multi-color emission capabilities, namely as an imaging agent within a hybrid system. An example of this is presented by Liu et al., where carbon dots are integrated into the core of a mesoporous silica nanoparticle, then functionalized with rose bengal as the photosensitizing agent and ampicillin for an added antibacterial mechanism [5]. Due to the nature of the particle created, the authors specify that no interactions between the carbon dots and rose bengal occur, unlike for the PpIX hydrogel mentioned previously. The carbon dots therefore truly function solely as an imaging agent in this photodynamic hybrid structure.

### 4.3. Carbon Nanodots in Photodynamic Antimicrobial Materials

**Figure 13:**
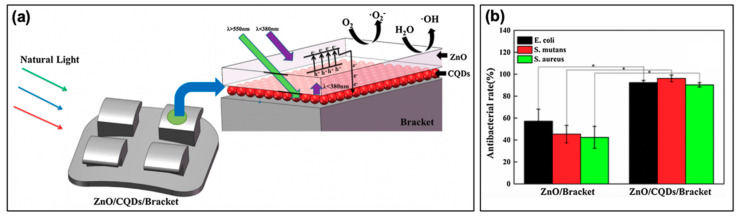
Carbon nanodots in materials applications. (**a**) Schematic for a zinc oxide (ZnO) and carbon nanodot (CQD) antimicrobial bracket coating for orthodontics materials. The schematic displays the reactive oxygen species (ROS, •O_2_^−^, •OH) generated by ultraviolet (λ < 380 nm) activation of the ZnO coating, enhanced both by the up-conversion properties (λ_ex_ > 550 nm → λ_em_ < 380 nm) and improved separation of electron/hole (e^−^/h^+^) pairs resulting from carbon dot incorporation. (**b**) Antibacterial efficiency of the bracket coatings under natural light irradiation for multiple bacterial strains; * *p* < 0.01. For additional details regarding the reported studies, the reader is referred to Table 2 of the main text. **Permissions***:* Reprinted from Chemical Physics Letters, 706, Zhang, J. et al., “Enhanced antibacterial properties of the bracket under natural light via decoration with ZnO/carbon quantum dots composite coating”, 702–707, Copyright © 2018, with permission from Elsevier [40].

Many of these early studies of carbon dots as photosensitizers for APDT or photocatalytic disinfection have not yet incorporated the structures into materials which may be more practically applied commercially and industrially; however, this materials development and focus on application is not absent from the current literature. A number of studies so far have been mentioned, including the CDs/ZnO bracket coating [40]. A schematic for this material is presented in Figure 13a. Brackets are commonly used orthodontics technology which presents a major challenge in regard to disinfection. Bacterial growth on brackets can result in enamel demineralization, which can ultimately lead to tooth decay and severe infection. To combat this, Zhang et al. created a carbon dot/zinc oxide hybrid coating to place on the surface of a metal bracket. Zinc oxide itself generates e^−^/h^+^ pairs capable of producing ROS as described previously. This material suffers, however, as it operates optimally with UV excitation. This is particularly problematic for an orthodontic application, as UV light is quite carcinogenic to biological tissues. During sterilization treatments, it would be extremely challenging if not exceedingly impractical to focus UV specifically to the bracket, rather than surrounding oral tissues. To overcome this limitation, the carbon dots were incorporated (additional carbon dot characterization can be found in Ref. [74]). As discussed earlier, this resulted in an increased visible light absorption from the coating as compared to the ZnO alone. Subsequent up-conversion luminescence from the carbon dots improved the absorption efficiency and ROS generation capabilities of the hybrid material from natural light, rather than ultraviolet, excitation. Accordingly, the antibacterial efficacy was examined, and it was found that efficiency was improved for *E. coli, Streptococcus mutans (S. mutans),* and *S. aureus* strains relative to the ZnO/Bracket material (Figure 13b) [40].

Although this study could benefit from deeper analysis into bracket surface morphology changes, long term efficacy, and biofilm formation, it sets a promising foundation for the use of carbon dots in hybrid materials for APDT and photocatalytic applications. Additional studies in this vein include the previously discussed CD-DNA-PpIX hydrogel [59], the Langmuir-Blodgett thin films of hydrophobic carbon quantum dots [36], and the CD@TiO_2_ polyester materials [27].

## 5. Practical Considerations

Before carbon dots can truly see pervasive use in APDT and photocatalytic disinfection applications, additional considerations and challenges must be addressed. Cytotoxicity and characterization complexities of carbon dots are both included herein, although these two factors are by no means exhaustive. These aspects will be discussed briefly in this section.

### 5.1. Cytotoxicity of Carbon Nanodots

**Figure 14:**
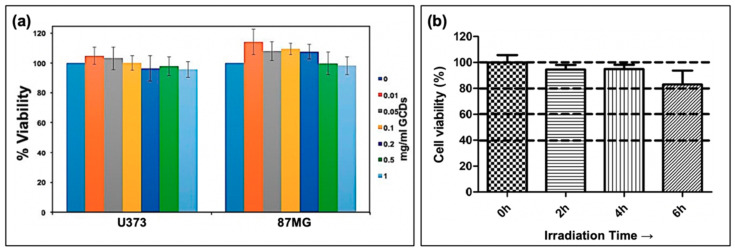
Example cytotoxicity data from carbon nanodot samples. (**a**) Concentration-dependent viability of Glioblastoma cell lines (U373 and 87MG) treated under dark conditions with carbon nanodots (GCD) at varying concentrations. Results demonstrate no significant cytotoxicity from carbon nanodot exposure. (**b**) Viability of NIH/3T3 cells exposed to carbon nanodots under light irradiation intervals. Results demonstrate the condition-dependent cytotoxicity of carbon nanodots. For additional details regarding the reported studies, the reader is referred to Table 2 of the main text. **Permissions***:* (**a**) Adapted with permission from Reference [22]. Copyright © 2019, American Chemical Society. (**b**) Adapted with permission from Reference [36]. Copyright © 2018, American Chemical Society.

In both APDT and photocatalytic disinfection applications there is potential for contact between mammalian cells or tissues and the agents of photosensitization. For APDT in particular this is an inevitable aspect, as treatments are applied once infections have already taken hold in the patient. In terms of photocatalytic disinfection, however, this is a no less important consideration. Although for many future developed disinfection materials the carbon dot systems might be fixed and stable, it is also possible that solution-based reagents for disinfection may constitute an application strategy for these nanoparticles. Solution-bound agents, unfixed to a solid substrate, thus have a higher chance of transferring from surfaces to biological systems, thereby presenting a potential health challenge. To date, carbon nanodots have been investigated for their cytotoxicity to mammalian cells; largely, they are reported as non-toxic at reasonable concentrations both in vitro and in vivo [92,93,94] although they have been identified to trigger off-target responses, such as inflammatory behavior, dependent on structural/chemical properties [95]. Particularly for the APDT literature for carbon dots, cytotoxicity is only sometimes probed and discussed, with researchers largely citing other carbon dot reports for verification of their benign nature. This is not necessarily adequate, however, due to the extreme variability that is present in carbon dot samples yielded from different labs by different synthetic procedures and purification methods. An example case where the researchers did in fact probe the cytotoxicity in conjunction with APDT effects is presented in Figure 14a.

In this report, Thakur et al. synthesized carbon dots from *Citrus limetta* waste pulp, producing particles (GCDs) that exhibited antibacterial activity against *E. coli* and *S. aureus* [22]. They then tested the cytotoxicity of these particles against U373 and 87MG Glioblastoma cell lines using a CKK8 assay; the results are presenting in Figure 14a. For both cell lines, there was no significant toxicity detected with up to 1 mg/mL concentration of carbon dots. While this is promising, there are some challenges in the comparison of these to the antibacterial data. For example, in the disk diffusion assays used to probe APDT, concentrations of carbon dots were reported between 1–600 mM, rather than in mg/mL, and with visible light irradiation. For the cytotoxicity study, the authors did not indicate whether or not the photosensitization mechanism was tested during the cell line exposure [22]. This is an important consideration, particularly for topical APDT applications, although it is not uncommon that light-exposed conditions are neglected in cytotoxicity characterization [5,12]. If a material generates ROS that do not act specifically on the bacterial cells instead of nearby mammalian cells, cellular damage and tissue damage could occur during treatment. Although this effect could be minimal depending on the characteristics of the material in question, it still merits investigation and reporting as the field develops. An example where carbon dot photosensitizers *do* lead to cytotoxicity is presented in Figure 14b [36]. For this study, NIH-373 mouse embryonic fibroblast cells were tested with irradiation intervals of varying length. After 6 h irradiation, toxicity was observed, although there were no reported changes in treated cell morphologies. It is noted by the authors that bacterial strains and the NIH-373 cell lines have different mechanisms to cope with oxidative stress, which could be promising in helping to minimize cytotoxicity in APDT-based treatment materials. This cell line was also studied in another report [49]. Additional cell lines investigated include mammalian HeLa cells [17,19], human embryo lung fibroblast (HELF) cells [5], V79 [12], and HepG2 cells [12], to name a few. More thorough characterization of the cytotoxicity of carbon dots against these cells and others should be undertaken as these materials are developed.

### 5.2. Carbon Nanodot Definition and Characterization

A second challenge facing the application of carbon dots as APDT photosensitizers is a fundamental one. Although characterization methods and common structural properties were outlined in Section 2, not all studies which report the synthesis of carbon dots reflect the properties mentioned. For example, carbon dots are typically noted as <10 nm in size, however, instances even discussed herein provide sizes much greater than this [49]. The morphology of carbon dots is also commonly accepted as spherical, yet this is not always the case, with heights reported for “carbon dot” structures that indicate perhaps only 1 or 2 stacked graphene sheets [25,36]; these structures, in fact, are more closely aligned with “graphene quantum dots”, which have received their own due attention in the literature [31]. Furthermore, excitation-dependent emission is frequently a characteristic of carbon dots, yet researchers report structures with fixed emission profiles [3,5,17,22,49]. As mentioned, a possible source of excitation dependence in carbon dot samples is thought to arise from the inherent heterogeneity in chemical and structural makeup even within a single sample; this is the rationale provided by some researchers for excitation-independent carbon dot samples following purification [55]. Yet still, other studies point to multiple discrete electronic states or heterogeneous surface states within a carbon dot structure as the source of excitation-dependence [17,96,97]. This is clearly still a point of discussion in the literature, that will require further elucidation or possibly descriptive re-framing in the accepted definition of carbon dots. In general, current characterization of surface charge is also typically weak. As recent reports suggest, this is a major factor in determining the efficacy of APDT and must be incorporated into the materials characterization effort moving forward. While carbon dots hold immense promise for these applications, further characterization and a widely accepted definition of the “carbon dot” structure is indeed required.

## 6. Conclusions and Perspectives

Herein we have provided a discussion of the current literature for carbon nanodots as photosensitizers for antimicrobial photodynamic therapies and photocatalytic disinfection. Although still an evolving field, the publications reviewed herein set the foundation for future growth and optimization of carbon nanodots with intrinsic photodynamic antibacterial capabilities. By tuning properties such as fluorescence or phosphorescence quantum yield, precursors and passivation agents, surface charge and more, still more effective antibacterial and antibiofouling materials may yet be achieved using carbon nanodots as a scaffold. Furthermore, hybrid materials incorporating carbon dots both with or without intrinsic antibacterial activity show immense promise in the development and optimization of new, inexpensive, and potentially even “green” materials. Still, we propose deeper characterization and methodical examination of trends for structural/chemical tuning, selectivity and activity against strains of differing Gram stains, carbon dot photosensitization-induced cytotoxicity, characterization of carbon dots themselves, and more. In all, the research reviewed herein provides a solid foundation for the future pursuit, characterization, and development of these carbon nanodot materials for APDT and photocatalytic disinfection applications.

## Figures and Tables

**Table 1:** Summary of Experimental Parameters for Studies Appearing in Figures for Section 3: Carbon Nanodots as Photodynamic Antimicrobial Agents.

This Work		Parameters								Ref. Details	
Figure	Panel	CND Label ^a^	Diameter	Charge ^b^	[CND]	Source	Power	Distance	Time	Ref.	Figure
3	b	CDs/Na2W4O13/WO3 photocatalyst	~6 nm	n.d.	1 mg/mL photocatalyst	visible light (>400 nm)	300 W Xe lamp	n.d.	0–100 min	[12]	7
3	c	brominated CND	~5 nm	negative	n.d.	365 nm	100 W lamp	~20 cm	4 min	[56]	3a
3	d	(T_1_) C-dots	~1.6 nm	n.d.	n.d.	365 nm	3V, 3W LED	n.d.	RT	[55]	2d
3	e	LB hydrophobic CQDs thin film	5 nm/~1 nm (height)	n.d.	n.d.	470 nm	15 W	50 cm *	2 h	[36]	4c
4	a (top)	brominated CND	~5 nm	negative	n.d.	365 nm	3.0 ± 0.1 mW	~15 cm	5 min	[56]	6a
4	a (middle)	brominated CND	~5 nm	negative	n.d.	N/A	N/A	N/A	N/A	[56]	6b
4	a (bottom)	brominated CND	~5 nm	negative	n.d.	365 nm	3.0 ± 0.1 mW	~15 cm	5 min	[56]	6c
4	b	brominated CND	~5 nm	negative	n.d.	365 nm	3.0 ±. 0.1 mW	~15 cm	5 min	[56]	6d
4	c	EDA-Cdots	~5 nm	positive ^†^ [21]	n.d.	visible light	12V 36W bulb	n.d.	0-8 h	[26]	5
5	a	EDA-Cdots	4–5 nm	positive ^†^ [21]	5 μg/mL	white light bulb	36W	10 cm	1 h	[16]	4a,b
5	b (left)	Cdots (from date palm fronds)	~50 nm	neutral	70 μg/mL	room light	50 W LED	8 ft ceiling	0 h	[49]	8b
5	b (right)	Cdots (from date palm fronds)	~50 nm	neutral	70 μg/mL	room light	50 W LED	8 ft ceiling	10 h	[49]	8c
6	b	EDA-Cdots	< 5 nm	positive ^†^ [21]	15.8 μg/mL	visible (“lab”) light	12V 36W LED	10 cm	1, 3 h	[24]	3a
6	c (left)	(T_1_) C-dots in PVA matrix	~1.6 nm	n.d.	0.1 μg/mL	UV *	n.d.	n.d.	N/A	[55]	3a
6	c (right)	(T_1_) C-dots	~1.6 nm	n.d.	n.d.	365 nm *	3V, 3W LED *	n.d.	Seconds *	[55]	3c
6	d (bottom)	“water dots”/”Br dots”	<10 nm	n.d.	n.d.	300 nm	n.d.	N/A	N/A	[46]	2b
6	d (top)	“water dots”/”Br dots”	<10 nm	n.d.	n.d.	300 nm	n.d.	N/A	N/A	[46]	2a
6	e	brominated CND	~5 nm	negative	n.d.	365 nm	3.0 ± 0.2 mW	~15 cm	4 min	[56]	7b
7	b	EDA-Cdots/EPA-Cdots	4–5 nm	EDA-(positive)/EPA-(neutral)	0.1–0.2 mg/mL	visible light (400–800 nm)	36W 12V bulb	10 cm	1 h	[21]	2
8	b	brominated CND	~5 nm	negative	n.d.	365 nm	~3 mW	~15 cm	4, 10 min	[56]	4b
8	c/d	brominated CND	~5 nm	negative	n.d.	365 nm	3.0 ± 0.2 mW	~15 cm	4 min	[56]	8a,b

* Denotes values extrapolated from relevant in-text details from the specified reference; † Denotes values extrapolated from related works (relevant reference indicated in brackets); ^a^ Labels indicate either additional details regarding the nature of the reported carbon nanodot or indicate the abbreviation/common label used within the cited study to describe the particle. ^b^ It must be noted that not all publications explicitly indicate the solution pH at which these measurements were made, a vital parameter in understanding particle charges.

**Table 2:** Summary of Experimental Parameters for Studies Appearing in Figures for Section 4 and Section 5.

This Work		Parameters								Ref. Details	
Figure	Panel	CND Label ^a^	Diameter	Charge ^b^	[CND]	Source	Power	Distance	Time	Ref.	Figure
9	a	EDA-Cdots	4–5 nm	positive ^†^ [21]	5 μg/mL	white light bulb	36 W	10 cm	1 h	[16]	1c
9	b	EDA-Cdots	4–5 nm	positive ^†^ [21]	0–64 μg/mL	visible light	12 V 36 W bulb	10 cm	1 h	[73]	3
9	c	brominated CND	n.d.	n.d.	n.d.	365 nm	100 W lamp	~20 cm	4 min	[60]	5b
10	a	CDs/CDs-Penicillin/PCDs	4 nm/47 nm/5 nm	n.d.	30 μg/mL	daylight	n.d.	n.d.	0–9 h	[19]	3c
10	b	BSA-CD NP/BSA-CD-Cipro NP	60–70 nm /~110–120 nm	-21.3 mV	1.47 μg/mL	Tungsten bulb (300–900 nm)	100 W	30 cm	1 h	[3]	6c
11	a/b	Cdots (from date palm fronds) with H_2_O_2_	~50 nm	neutral	9 μg/mL	sunlight	n.d.	n.d.	0–3 h	[49]	7a/b
11	c	CD/BSA-CD/BSA-CD NP	~5/8.5/60–70 nm	n.d./n.d. /−21.3 mV	n.d.	Tungsten bulb (300–900 nm)	100 W	30 cm *	0–120 min	[3]	5c
11	d	BSA-CD-Cipro NP	~110–120 nm	−21.3 mV	n.d.	N/A	N/A	N/A	0–60 h	[3]	5d
12	b	CD-DNA	~5 nm (pre-DNA)	negative *	n.d.	N/A	N/A	N/A	N/A	[59]	1b
12	c	CD-DNA	~5 nm (pre-DNA)	negative *	n.d.	410 nm	N/A	N/A	N/A	[59]	1c
12	e	ZnO/CQDs/Bracket	~7 nm ^†^ [74]	n.d.	1.5 mg/mL (pre-coating)	550–850 nm	N/A	N/A	N/A	[40]	6b
12	f	ZnO/CQDs/Bracket	~7 nm ^†^ [74]	n.d.	1.5 mg/mL (pre-coating)	N/A	N/A	N/A	N/A	[40]	5
13	b	ZnO/CQDs/Bracket	~7 nm ^†^ [74]	n.d.	1.5 mg/mL (pre-coating)	natural light	n.d.	n.d.	24 h	[40]	4d
14	a	LB hydrophobic CQDs thin film	5 nm/~1 nm (height)	n.d.	n.d.	470 nm	15 W	50 cm *	0–6 h	[36]	8
14	b	“green carbon dots” (GCD)	4–7 nm	n.d.	0.01–1 mg/mL	dark conditions	N/A	N/A	24 h	[22]	6b

* Denotes values extrapolated from relevant in-text details from the specified reference; † Denotes values extrapolated from related works (reference indicated in brackets); ^a^ Labels indicate either additional details regarding the nature of the reported carbon nanodot or indicate the abbreviation/common label used within the cited study to describe the particle. ^b^ It must be noted that not all publications explicitly indicate the solution pH at which these measurements were made, a vital parameter in understanding particle charges.
